# Two New Pentadepsipeptides from the Mangrove Fungus *Aspergillus* sp. SCSIO 41443

**DOI:** 10.3390/metabo16030159

**Published:** 2026-02-27

**Authors:** Ying Liu, Yi Chen, Jiao Xiao, Xin Sun, Xuefeng Zhou, Yonghong Liu, Bin Yang

**Affiliations:** 1 Wuya College of Innovation, Shenyang Pharmaceutical University, Shenyang 110016, China; 15248089565@163.com (Y.L.); yonghongliu@scsio.ac.cn (Y.L.); 2 State Key Laboratory of Tropical Oceanography/Guangdong Key Laboratory of Marine Materia Medica, South China Sea Institute of Oceanology, Chinese Academy of Sciences, Guangzhou 510301, China; chenyi221@mails.ucas.ac.cn (Y.C.); 18844971326@163.com (X.S.); xfzhou@scsio.ac.cn (X.Z.); 3 University of Chinese Academy of Sciences, Beijing 100049, China; 4 Shenzhen Clinical College of Integrated Chinese and Western Medicine, Guangzhou University of Chinese Medicine, Shenzhen 518104, China

**Keywords:** mangroves, *Aspergillus* sp., pentadepsipeptides, structures

## Abstract

**Background**: Mangrove fungi are a prolific source of structurally diverse natural products. Among these, natural peptides with varied biological activities hold high commercial value and have been successfully developed into drugs for treating numerous diseases. **Methods**: Following rice solid-state fermentation of the strain, extracellular metabolites were extracted from the culture filtrate to obtain a crude extract. Cyclic depsipeptides were isolated from the crude extract by silica gel vacuum liquid chromatography for preliminary fractionation and enrichment, followed by high-performance liquid chromatography (HPLC) purification. The structures of the compounds were determined based on extensive spectroscopic analysis (1D and 2D NMR, ESI-MS-MS analysis) and Marfey’s method for amino acid configuration assignment. **Results**: Ultimately, two new compounds, aspertides F (**1**) and G (**2**), and three known compounds, aspertides C, D, and A (**3**−**5**), were identified. The bioassays indicated that these compounds exhibited weak activity against acetylcholinesterase and neuraminidase. **Conclusions:** The research findings on this strain have not only enriched the metabolic resource library of mangrove fungi but also highlighted their diverse biological activities and significant application potential.

## 1. Introduction

Mangrove forests represent a typical extreme intertidal environment that fosters rich and diverse microbial communities [1]. Unlike most ecosystems, mangroves are characterized by tidal action, high salinity, and anoxic soils [2]; these conditions exert intense natural selection pressure on microorganisms, driving the evolution of unique and efficient metabolic pathways. The microorganisms isolated from mangrove samples encompass multiple genera of fungi, bacteria, and other groups, among which Aspergillus fungi represent one of the most extensively studied and representative taxa. However, international research on microbial natural products derived from Sri Lankan mangroves remains extremely limited, with scarce information available regarding their chemical profiles and biological activities. Therefore, this study focuses on regionally distinctive strains from this unique ecological niche, aiming not only to explore potentially novel compounds within their metabolic profiles but also to elucidate the metabolic potential and adaptive strategies of microorganisms in this specific habitat from the perspective of chemical diversity. The work is expected to provide a new, ecologically informed, and regionally distinctive direction for natural product discovery.

At present, a wealth of structurally novel metabolites has been identified from marine *Aspergillus* fungi. Based on their structures, these compounds are mainly classified into butenolides [3], alkaloids [4], terpenes [5], polyketides [6], and cyclic peptides [7], among others, and exhibit various biological activities such as antibacterial [8], anti-inflammatory [9], and antitumor [10] effects. The dimeric compound brevianamide S [11] isolated from *Aspergillus versicolor* exhibits significant antibacterial activity against *Bacille Calmette-Guerin* (BCG). Variecolortins B and C [12], a pair of enantiomers isolated from the marine sediment-derived fungus *Eurotium* sp. SCSIO F452, exhibit distinct antioxidant and cytotoxic activities. The study of *Aspergillus* secondary metabolites expands the natural product database and provides molecular resources for marine drug development.

**Figure 1:**
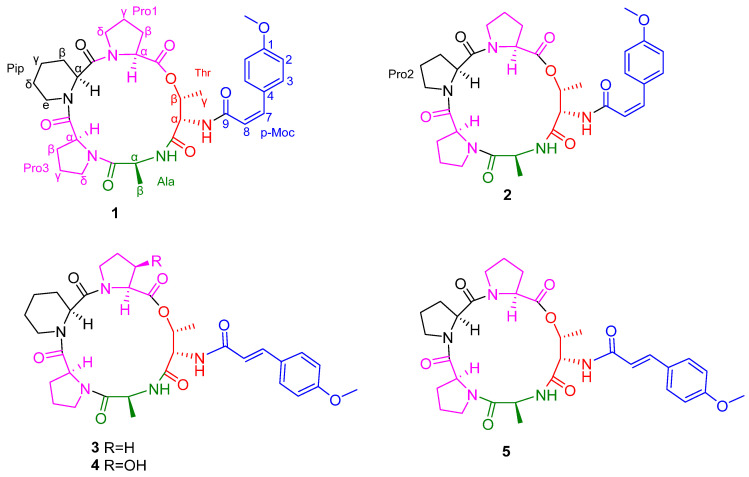
Structures of compounds **1**−**5**.

Among them, peptides are important and unique natural products that contain both ester and amide bonds. They exist in many marine microorganisms and are closely related to the biosynthesis of nonribosomal peptide synthetases [13]. Most of the reported peptides show good antibacterial activity [14,15], which makes them promising compounds for antibiotic discovery. In this study, we focused our research on the strain *Aspergillus* sp. SCSIO 41443. As shown in Figure 1, two new cyclic peptide compounds, named aspertides F (**1**) and G (**2**), together with three known peptides, aspertides C, D, and A (**3**−**5**) [16], from the extract of the strain cultured in rice medium. This study reports the fermentation, isolation, structural elucidation, and bioassay screening of these compounds.

## 2. Materials and Methods

### 2.1. General Experimental Procedures

UV spectra were recorded on a UV-2600 PC spectrometer (Shimadzu, Beijing, China). IR spectra were acquired on an IR Affinity-1 spectrometer (Shimadzu, Beijing, China). Optical rotations were measured on an Anton Paar MPC500 polarimeter (Anton, Graz, Austria). Circular Dichroism (CD) spectra were measured on a Chirascan spectrometer (Applied Photophysics, Leatherhead, UK). NMR spectra were acquired with tetramethylsilane as the internal standard, using the following spectrometers: a Quantum-I Plus operating at 500 MHz for ^1^H and 125 MHz for ^13^C (Q-one Instrument Co., Ltd., Wuhan, China); and an AVANCE III HD operating at 700 MHz for ^1^H and 175 MHz for ^13^C (Bruker Switzerland AG, Fällanden, Switzerland). High-resolution electrospray ionization mass spectrometry (HR-ESI-MS) was performed using a Bruker maXis Q-TOF instrument (Bruker BioSpin International AG, Fällanden, Switzerland). Semipreparative high-performance liquid chromatography (HPLC) separation was carried out using the Hitachi Primaide system (Hitachi, Tokyo, Japan) equipped with a DAD detector and an ODS column (ChromCore 120 C18, 10 × 250 mm, 5 μm). Rotary evaporator column chromatography (CC) was carried out with silica gel (200−300 mesh; Qingdao Marine Chemical Factory, Qingdao, China) and ODS (50 μm; Merck, Rahway, NJ, USA). For column chromatography, methanol, ethyl acetate, petroleum ether, and dichloromethane (analytically pure, Tianjin Damao Chemical Reagent Factory, Tianjin, China) were employed as eluents. Medium-pressure preparative chromatography was performed on an LC3000 system (BÜCHI Labortechnik AG, Flawil, Switzerland). Ultrasonic treatment was performed using a KQ-250DB ultrasonic cleaner (Yuhua Instrument Co., Ltd., Gongyi, China). Rotary evaporation was carried out on an EYELAN-1001 rotary evaporator (Ailang Instrument Co., Ltd., Shanghai, China). Vacuum was provided by an SHZ-CB circulating water vacuum pump (Yuhua Instrument Co., Ltd., Gongyi, China).

### 2.2. Statistical Analysis and Data Processing (Enzyme-Inhibitory Activity)

In the bioactivity assays, the reagents used also included phosphate-buffered saline (PBS, pH 7.4) (Sersvicebio, Wuhan, China), 5,5′-Dithio bis-(2-nitrobenzoic acid) (DTNB) (Macklin, Shanghai, China), acetylthiocholine iodide (Sigma-Aldrich Co., St. Louis, MO, USA), acetylcholinesterase (Macklin, Shandong, China), tacrine (Macklin, Shandong, China), and a neuraminidase inhibitor screening kit (Beyotime, Shanghai, China). The absorbance was measured at the required wavelength using a microplate reader (Thermo Fisher Multiskan FC, Waltham, MA, USA).

Statistical analysis and data visualization were performed using Origin 2024. Molecular docking simulations were carried out with AutoDock 4.2.6 (The Scripps Research Institute, San Diego, CA, USA). Protein and ligand samples were prepared using AutoDock Tools 1.5.7 (The Scripps Research Institute, San Diego, CA, USA), and the resultant structures were visualized via PyMOL 3.1.3 (Schrödinger, LLC, New York, NY, USA).

### 2.3. Isolation, Cultivation, and Identification of Fungal Aspergillus Strains

The fungal strain *Aspergillus* sp. SCSIO 41443 was obtained from sediment samples collected at Mangrove Park (NARA), Sri Lanka. This strain is maintained at the CAS Key Laboratory of Tropical Marine Bioresources and Ecology, South China Sea Institute of Oceanology, Chinese Academy of Sciences, Guangzhou, China. Based on Basic Local Alignment Search Tool (BLAST, ver.2.14.1+) analysis of the Internal Transcribed Spacer (ITS) sequence (Supplementary Materials), which showed 98% similarity to *Aspergillus* tamarii, the strain was designated as *Aspergillus* sp. SCSIO 41443. The sequence was deposited in GenBank with the accession number PX915610.

Strain identification was performed by Beijing Tsingke Biotechnology Co., Ltd., Beijing, China.

### 2.4. Fermentation, Extraction, Isolation, and Purification

The strain *Aspergillus* sp. SCSIO 41443 was statically cultivated on Marine Agar (MA) medium and then was cultured in 200 mL seed medium (1.5% malt extract, 2.0% sea salt) in 1 L Erlenmeyer flasks at 26 °C for 3 days on a rotary shaker (180 rpm). A large-scale fermentation was conducted in 48 units of 1L flasks containing a rice medium (150 g rice, 3.2% sea salt, and 150 mL H_2_O). The cultures were incubated statically at 26 °C for 28 days. Fungal mycelia and culture medium were blended with ethyl acetate (EtOAc) in a 1:1 (*v*/*v*) ratio, followed by ultrasound extraction for 20 min. After extraction, the mixture was filtered to remove fungal mycelial biomass, and the organic phase was separated. After three repeated extractions, the combined organic supernatants were rotary-evaporated to dryness, yielding 63 g (*d*/*w*) of crude extract.

The ethyl acetate extract was pretreated by mixing with silica gel (300−400 mesh) in a 1:1 (*w*/*w*) ratio. Subsequently, based on a 1:3 (*w*/*w*) mass ratio of the sample to the stationary phase (100−200 mesh silica gel), the silica was weighed for dry packing of the column. The pre-adsorbed sample was then loaded onto the column and fractionated via vacuum liquid chromatography (VLC) using a gradient elution method. The separation was achieved using a step gradient elution program. The column was first eluted with petroleum ether (PE)-dichloromethane (DCM) (ν:ν 1:0, 3:1, 1:1, 1:3, 0:1) and DCM-methyl alcohol (CH_3_OH) (ν:ν 100:1, 50:1, 100:3, 20:1, 5:1, 2:1, 0:1). The entire elution process was monitored by analytical thin-layer chromatography (TLC) under UV light (254 nm). Based on the similarity of TLC profiles (Rf values, spot patterns), the collected eluates were pooled, resulting in a total of 14 fractions (Frs. 1−14). Fr. 10 to Fr. 12 were merged and then purified on an ODS silica gel column. Using a CH_3_OH/H_2_O gradient (5−100%) as the eluent, 6 subfractions were obtained, designated as Frs. 10-1 to 10-6. Based on this, Fr. 10-4 was purified by semipreparative HPLC (60% CH_3_CN/H_2_O, 3.0 mL/min), yielding three compounds: compound **3** (9.47 mg, t_R_ 11.5 min), compound **4** (6.76 mg, t_R_ 19.5 min), and compound **5** (9.61 mg, t_R_ 15.7 min). Compound **1** (1.69 mg, t_R_ 13.2 min) was purified from Fr. 10-4-3 by semipreparative HPLC (40% CH_3_CN/H_2_O, 2.5 mL/min). Similarly, compound **2** (2.33 mg, t_R_ 17.5 min) was obtained from Fr. 10-4-5 under the same chromatographic conditions.

### 2.5. Amino Acid Hydrolysis and Chiral HPLC Analysis

The hydrolysis and derivatization procedures were performed following a previously described method [17], with modifications. Briefly, compounds **1** and **2** (0.5 mg each) were, respectively, hydrolyzed with 1 mL of 6 M HCl at 115 °C for 24 h. After cooling, the solution was evaporated to dryness. The resulting hydrolysate was then mixed with 100 μL of H_2_O, 100 μL of 1% FDAA (Marfey’s reagent, 1-fluoro-2,4-dinitrophenyl-5-L alanine amide) in acetone, and 20 μL of 1 M NaHCO_3_. The reaction mixture was incubated at 40 °C for 2 h and subsequently quenched by adding 20 μL of 1 M HCl. For comparison, standard amino acids _D/L_-proline (Pro), _D/L_-alanine (Ala), _D/L_-pipecolinic (Pip), _D/L_-threonine (Thr) were derivatized with FDAA using the same procedure. The dried mixture was dissolved in CH_3_CN and analyzed by HPLC on the Hitachi Primaide system equipped with a DAD detector (Hitachi, Tokyo, Japan). The separation was achieved using a Nano ChromCore C18 column (5 μm, 250 × 10 nm) with a mobile phase consisting of CH_3_CN and H_2_O (0.04% formic acid). A linear gradient starting from 15% to 55% CH_3_CN over 55 min was applied at a flow rate of 2 mL/min. Finally, the retention times of the FDAA derivatives of compounds **1**−**2** were compared with those of the derivatized standard amino acids (see Figures S22 and S23).

### 2.6. Acetylcholinesterase Inhibitory Activity

Based on a molecular networking screening, compounds **1**−**5** were chosen for subsequent evaluation of their inhibitory activity against AChE. In vitro experiments were performed using a 96-well plate method according to the modified Ellman assay [18]. AChE (U denotes enzyme activity units) was prepared as a 0.1 U/mL working solution in potassium PBS, with a final reaction concentration of 0.02 U/mL; the final concentration of compounds **1**−**5** was 50 μg/mL. After incubating the 96-well plate at 30 °C for 20 min, a mixture of acetylthiocholine iodide and DTNB (mixed in equal volumes immediately before use) was added to achieve a final reaction concentration of 0.625 mM. The reaction system was incubated at 30 °C for 30 min, and absorbance was measured at a wavelength of 405 nm using a microplate reader, with tacrine serving as the positive control.

All assays were performed in triplicate in 96-well microplates, with separate groups including the test sample group, negative control group (DMSO solvent), positive control group (tacrine), and background control group without enzyme. The inhibition rate was calculated after correction for background absorbance according to the following formula:

$$Inhibition\;rate\;(\%)=\;\frac{\left(B-B_{0}\right)-(A-A_{0})}{B-B_{0}}\times 100\%$$

where A and A_0_ represent the absorbance values of the test group and its background control, respectively, and B and B_0_ denote those of the negative control group and its corresponding blank control, respectively. Compounds with an inhibition rate higher than 10% were considered to exhibit significant AChE inhibitory activity.

### 2.7. Neuraminidase Inhibitory Activity

The inhibitory activities of compounds **1**–**5** were evaluated using a neuraminidase inhibitor screening kit. Test samples were prepared at a concentration of 0.5 mg/mL. Briefly, neuraminidase assay buffer, neuraminidase, and Milli-Q water were sequentially added to a 96-well plate according to the manufacturer’s instructions. The mixture was gently mixed and incubated at 37 °C for 2 min to allow sufficient interaction between the inhibitors and neuraminidase. Subsequently, the neuraminidase fluorescent substrate was added to a final volume of 100 μL. After incubation at 37 °C for 30 min, the fluorescence intensity of the reaction mixture was measured with a multifunctional microplate reader at an excitation wavelength of 322 nm and an emission wavelength of 450 nm. The inhibition rate of samples against neuraminidase was calculated based on the standard curve. For compounds showing inhibitory activity in the screening assay, the half-maximal inhibitory concentration (IC_50_) was determined via dose–response experiments.

### 2.8. Molecular Docking

Molecular docking was performed using AutoDock 4.2.6 [19]. First, the crystal structure of acetylcholinesterase [20] (PDB code: 4EY7) was preprocessed in AutoDock Tools 1.5.7 by adding hydrogen atoms, assigning partial charges, and defining atom types. A semi-flexible docking protocol was then applied to dock the target compounds into the enzyme’s active site. All structural visualizations were prepared using PyMOL 3.1.3.

## 3. Results

### 3.1. Spectroscopic Data of Compounds

Aspertide F (**1**). White Powder; [*α*${]}_{D}^{25}$ −17.5 (*c* 0.1, CH_3_OH); UV (CH_3_OH) *λ*_max_ (log *ε*) 200 (1.60), 247 (0.25), 302 (0.45) nm (Figure S10, Supplementary Materials); CD (0.3 mg/mL, CH_3_OH) *λ*_max_ (Δ*ε*) 200 (8.77), 211 (−15.19), 243 (+0.28) nm (Figure S11, Supplementary Materials); IR (film) *ν*_max_ 3334, 1645, 1014, 678, 599 cm^−1^ (Figure S9, Supplementary Materials); ^1^H and ^13^C NMR data, Table 1; ESI-MS/MS (Figure S8, Supplementary Materials): *m/z* 638.3182 [M+H]^+^, 541.2656 [Pip+Pro+Ala+Thr-p-Moc+H]^+^, 430.1971 [Pro+Ala+Thr-p-Moc+H]^+^, 355.1263 [Ala+Thr-p-Moc+Na]^+^, 280.1654 [Thr-p-Moc+Na]^+^; HR-ESI-MS (Figure S7, Supplementary Materials): *m/z* 638.3208 [M+H]^+^ (calcd for C_33_H_44_N_5_O_8_, 638.3208).

Aspertide G (**2**). White Powder; [*α*${]}_{D}^{25}$ −12.7 (*c* 0.1, CH_3_OH); UV (CH_3_OH) *λ*_max_ (log *ε*) 200 (1.75), 248 (0.19), 291 (0.38) nm (Figure S20, Supplementary Materials); CD (0.3 mg/mL, CH_3_OH) *λ*_max_ (Δ*ε*) 200 (13.54), 223 (−9.06), 278 (+1.66) nm (Figure S21, Supplementary Materials); IR (film) *ν*_max_ 3402, 1635, 1018, 698 cm^−1^ (Figure S19, Supplementary Materials); ^1^H and ^13^C NMR data, Table 1; HR-ESI-MS(Figure S18, Supplementary Materials): *m/z* 624.3031 [M+H]^+^ (calcd for C_32_H_42_N_5_O_8_, 624.3031).

### 3.2. Fermentation Yields of Target Compounds

In this study, compounds **1**–**5** were isolated and purified from the combined starting fractions 10–12 (approximately 2 g) by ODS reversed-phase column chromatography and preparative HPLC. Their masses were determined to be 1.69, 2.33, 9.47, 6.76, and 9.61 mg, respectively. Based on the dry weight of the fermented mycelial biomass (63.1 g), the relative productivities of these compounds were calculated as 0.027, 0.037, 0.150, 0.107, and 0.152 mg/g dry mycelium, respectively. These results indicate low fermentation yields of the target compounds, which represent a limitation of the present work.

### 3.3. Structural Elucidation and Identification of Pentadepsipeptides

**Figure 2:**
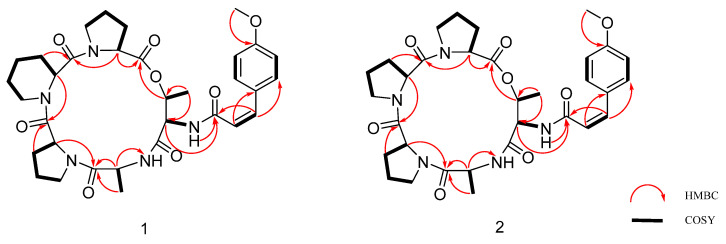
Key HMBC and ^1^H-^1^H COSY correlations for compounds **1** and **2**.

**Figure 3:**
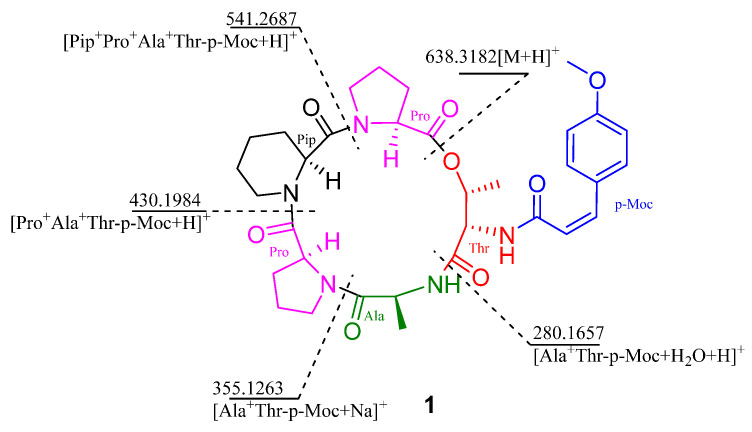
ESI-MS/MS analysis of compound **1**.

Compound **1** was determined to have the molecular formula C_33_H_43_N_5_O_8_ based on HR-ESI-MS data, implying 15 degrees of unsaturation. The NMR spectra exhibited characteristic signals of a cyclic peptide. Analysis of the ^1^H, ^13^C, and 2D NMR (HSQC, HMBC, ^1^H-^1^H COSY, ROESY) data in Table 1 and Figures S1–S6 (Supplementary Materials) led to the identification of five amino acid residues: two Pro, a six-membered cyclic Pip, an Ala, and a Thr. The ^13^C NMR spectrum revealed six carbonyl carbons (δ_C_ 171.3, 171.3, 170.8, 170.7, 167.7, 165.9), five of which were assigned to peptide bonds, and one (δ_C_ 165.9) to an additional amide carbonyl. The ^1^H NMR spectrum displayed signals for a pair of ortho-coupled aromatic protons [δ_H_ 6.87 (d, *J* = 8.9 Hz, H-2,6) and 7.75 (d, *J* = 8.8 Hz, H-3, 5)], a pair of coupled olefinic protons [δ_H_ 6.66 (d, *J* = 13.0 Hz, H-7) and 6.14 (d, *J* = 12.9 Hz, H-8)], and a methoxy group (δ_H_ 3.76 and δ_C_ 55.1). These signals collectively indicated the presence of a p-methoxycinnamamide (p-Moc) moiety. A key HMBC correlation (Figure 2) from the amide carbonyl carbon of the p-Moc group (δ_C_ 165.9) to the amide proton (δ_H_ 7.71) attached to the Thr residue unambiguously located the p-Moc group on the N-terminus of Thr. Additionally, the HMBC correlations from the NH proton of the amino acid residue to the carbonyl carbon of the adjacent residue indicate that the amino acid residue sequence of compound **1** is cyclo-[Pro1–Pip–Pro3–Ala–Thr–p-Moc]. The cyclic structure was further supported by ESI-MS/MS analysis (Figure 3). Characteristic fragment ions at *m/z* 638.3182, 541.2656, 430.1971, 355.1263, and 280.1654 correspond to sequential ring-opening cleavages, consistent with the proposed sequence.

**Figure 4:**
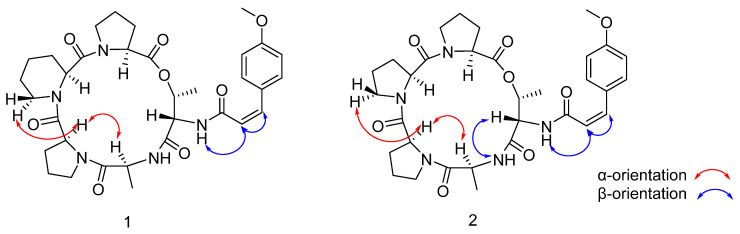
Key NOESY correlations of compounds **1** and **2**.

The NMR data for compound **1** were highly consistent with those reported for compound **3** (aspertide C) [16], and the main difference was the configuration of the olefinic bond. This conclusion is primarily supported by the observed vicinal coupling constant of 13.0 Hz between H-7 [δ_H_ 6.66 (d, *J* = 13.0 Hz)] and H-8 [δ_H_ 6.14 (d, *J* = 12.9 Hz)], which is characteristic of a cis-disubstituted alkene [21]. Comparative analysis of the NMR data, specifically the vicinal coupling constants between the olefinic protons, allowed for the definitive configurational assignment. The smaller coupling constant observed for compound **1** [δ_H_ 6.66 (d, *J* = 13.0 Hz), δ_H_ 6.14 (d, *J* = 12.9 Hz)] is indicative of a cis-double bond, whereas the larger constant for compound **3** [δ_H_ 7.37 (d, *J* = 15.8Hz), δ_H_ 6.94 (d, *J* = 15.8Hz)] is characteristic of a trans-double bond. The assignment was further corroborated by NOESY data (δ_H_ 6.66, 6.14) (Figure 4), which showed a correlation between these two protons, consistent with their cis stereochemical relationship. Through the advanced Marfey’s method and HPLC analysis (Figure S22, Supplementary Materials), the amino acid residues were identified as _L_-Pro, _L_-Pip, _L_-Ala, and _L_-Thr. In conclusion, the structure of compound **1** was unequivocally identified as cyclo-[_L_-Pro–_L_-Pip–_L_-Pro–_L_-Ala–_L_-Thr(p-Moc)]. Finally, compound **1** was identified as another new pentadepsipeptide and assigned the name aspertide F.

Compound **2** was determined to have the molecular formula C_32_H_41_N_5_O_8_ based on HR-ESI-MS data (see Supplementary Material Figure S18), implying 15 degrees of unsaturation. The ^1^H, ^13^C NMR (Table 1, Figures S12 and S13, Supplementary Materials), and 2D NMR (Figures S14–17, Supplementary Materials) spectra of compound **2** are very similar to those of compound **1**, except for the absence of the signals for Pro2 (*δ*_H_ 1.61/1.44; *δ*_C_ 18.33). The HMBC (Figure 2) correlation between *δ*_H_ 1.61/1.44 and δ_C_ 18.33 was used to locate the p-Moc group at the Thr NH. Furthermore, the sequential inter-residue HMBC correlations from the NH protons to the carbonyl carbons of the preceding residues established the cyclic sequence of compound **2** as cyclo-[Pro1–Pro2–Pro3–Ala–Thr–p-Moc]. According to the NOESY (Figure 4) and NMR dates, it was verified that the olefin signal was a cis-double bond, with minor differences in **5**. And the absolute configuration of amino acid residues was determined to be 3 _L_-Pro, 1 _L_-Ala, and 1 _L_-Thr by Marfey’s method and HPLC analysis (Figure S23, Supplementary Materials). Therefore, the structure of compound **2** was finally determined to be cyclo- [_L_-Pro–_L_-Pro–_L_-Pro–_L_-Ala–_L_-Thr (p-Moc)]. Finally, compound **2** was identified as another new pentadepsipeptide and assigned the name aspertide G.

Compounds **3**−**5** were identified to be the known aspertides C, D, and A [16] based on comparison of their ^1^H and ^13^C NMR data with literature values (Figures S24–29, Supplementary Materials).

### 3.4. Acetylcholinesterase Assay

**Figure 5:**
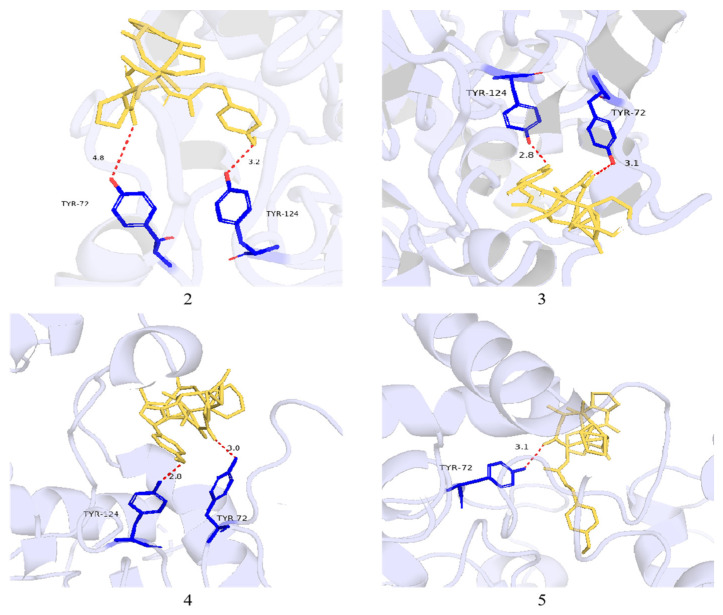
Proposed binding interactions of **2**−**5** with the active site residues of AChE (PDB ID: 4EY7). Red line: hydrogen bond.

Initial screening at 50 µg/mL identified compounds **1**–**5** as active neuraminidase inhibitors, with inhibition rates of 30.01%, 23.47%, 36.43%, 36.71%, and 27.42%, respectively (Figure 5, Figure S31, Supplementary Materials). Against a reference inhibition rate of 70.52% for tacrine, these compounds also exhibited moderate inhibitory activity against AChE at the same concentration, with inhibition rates of 22.03%, 24.67%, 23.19%, 20.26%, and 20.43%, respectively (Figure S30, Supplementary Materials). To further investigate their mechanism of action, the AChE inhibitory activity of **1**–**5** was evaluated by in silico molecular docking analysis.

Molecular interactions of the compounds were analyzed, and compounds **1**–**5** were selected for docking into the active site of AChE (PDB: 4EY7). The results showed that these molecules share similar binding positions, with binding free energy values (S value) all in the negative range, ranging from −8.51 to −10.13 kcal/mol. Compound **1** did not interact with the conventional active site residues; instead, compounds **2**–**5** primarily formed hydrogen bonds within the AChE active site. In particular, the methoxy group and carbonyl groups of compound **2** formed hydrogen bonds with Tyr 124 and Tyr 72, respectively. However, experimental assays revealed only weak activity, which is inconsistent with the docking results.

## 4. Discussion

Mangrove forests are typical extreme intertidal ecosystems. Environmental characteristics such as high salinity, tidal alternation, and anoxic soil exert strong selective pressure on microorganisms, driving the evolution of unique metabolic pathways. Marine-derived Aspergillus fungi have been proven to produce structurally diverse and biologically active metabolites, representing an important source for natural product research. However, studies on microbial natural products from Sri Lankan mangroves remain scarce worldwide. In this study, two new five-membered cyclic depsipeptides, aspertides F (**1**) and G (**2**), together with three known analogues, aspertides C, D, and A (**3**–**5**), were isolated from *Aspergillus* sp. SCSIO 41443, derived from mangrove sediments in this region. Structure elucidation and bioactivity screening were carried out. These results not only provide new data for the study of mangrove fungal metabolites but also reveal key issues to be addressed in the development of such compounds. The two new compounds isolated in this study, aspertides F (**1**) and G (**2**), are both five-membered cyclic depsipeptides bearing a p-Moc moiety, and their structural characteristics are highly similar to those of the known compound aspertide C (**3**). The core differences lie in the olefin bond configuration and amino acid residue composition: the p-Moc group of compound **1** features a cis-olefin bond, which is distinctly different from the trans-olefin bond in compound **3**. This configurational difference has been clearly verified by nuclear magnetic resonance coupling constant analysis and NOESY correlation experiments [21], thus enriching the structural diversity of five-membered cyclic depsipeptides derived from mangrove fungi.

In terms of biological activity, all compounds **1**−**5** exhibited inhibitory effects on AChE and neuraminidase at a concentration of 50 μg/mL, yet the overall activity was modest. The AChE inhibition rates were only 20.26% to 24.67%—markedly lower than the 70.52% AChE inhibition rate of tacrine, the positive control drug. To explore the underlying action mechanism, molecular docking simulations of the compounds with AChE were performed using AutoDock 4.2.6 [19]. The results demonstrated that all compounds formed interactions with the AChE active site with negative binding free energies, ranging from −8.51 to −10.13 kcal/mol. Notably, compounds **2**−**5** formed hydrogen bonds with key residues (e.g., Tyr 72 and Tyr 124) at the AChE active site, which theoretically indicated favorable binding affinity. However, a significant discrepancy was observed between these in silico results and the weak bioactivity detected in in vitro experiments. The fundamental cause of this inconsistency stems from the inherent difference between the static nature of molecular docking simulations and the dynamic complexity of in vivo biochemical systems [22,23]. These properties are pivotal determinants of the actual biological activity of compounds but are often underrepresented in standard docking scoring functions. This finding further corroborates the limitations of virtual screening in natural product research: virtual screening can only provide a theoretical framework for elucidating activity mechanisms and cannot serve as a substitute for in vitro experimental validation [24]. Future studies should integrate virtual screening with experimental validation to more accurately identify natural products with genuine biological activity.

This study also revealed that the fermentation yields of the target compounds were generally low, and such low productivity has become a major bottleneck restricting their further development and application. The yields of cyclic depsipeptide natural products are highly susceptible to fungal fermentation conditions. Based on previous studies, systematic optimization of fermentation parameters will be a feasible strategy to improve the yields of target compounds in future work [25,26]. Key efforts should focus on investigating the regulatory effects of medium composition (e.g., carbon sources, nitrogen sources, and sea salt concentrations) and fermentation temperature on the biosynthesis of pentadepsipeptides by *Aspergillus* sp. SCSIO 41443. Additionally, approaches such as chemical epigenetic modification and microbial co-cultivation can be explored to induce fungal metabolic pathways [6], thereby further enhancing the synthetic efficiency of the target products and providing a solid material basis for subsequent in-depth bioactivity studies and development.

From an application perspective, although the cyclic depsipeptides identified in this study exhibited only modest inhibitory activity against AChE and neuraminidase, they provide novel lead structures for the development of related enzyme inhibitors. AChE inhibitors are important therapeutic agents for Alzheimer’s disease, and neuraminidase inhibitors represent core drug targets for anti-influenza therapy. The cyclic depsipeptides discovered herein possess a unique structural scaffold incorporating a p-Moc group and a five-membered ring, which renders them promising lead compounds for structural modification and derivatization. Targeted modification of structural moieties, such as amino acid residues, olefin bond configurations, and substituents, is expected to optimize their binding affinity to enzyme active sites and improve their physicochemical properties. This approach may yield derivatives with significantly enhanced bioactivity, thereby providing a new direction for the development of novel enzyme inhibitors.

In summary, this study has enriched the metabolic resource library of pentadepsipeptides from mangrove fungi [7,16], clarified the structural characteristics and enzyme inhibitory activity potential of the target compounds, and revealed the core problems of weak biological activity and low fermentation yield for this class of compounds, as well as the complementarity between virtual screening and in vitro experimental validation in the activity evaluation of natural products [22,23,24]. Future research should focus on optimizing fermentation conditions to break through the yield bottleneck [25,26] and adopt targeted structural modification strategies to enhance the biological activity of the compounds [14,15], which provides important experimental evidence and research directions for the in-depth exploration of novel natural products from mangrove fungi and the study of their structure–activity relationships.

## 5. Conclusions

In summary, two new cyclic peptides, aspertides F and G (**1**−**2**), were isolated and identified from the mangrove sediment-derived fungus *Aspergillus* sp. SCSIO41443, along with three known cyclic peptides, aspertides C, D, and A (**3**−**5**). Based on NMR, HR-ESI-MS, and 2D NMR (HMBC, HMQC, COSY) data, combined with acid hydrolysis and chiral high-performance liquid chromatography (HPLC) analyses, the structures and absolute configurations of compounds **1**−**5** were elucidated. The potential of this compound as an acetylcholinesterase inhibitor was evaluated using an integrated approach combining virtual screening and experimental validation; however, its inhibitory efficacy was limited. Bioassay data for acetylcholinesterase and neuraminidase inhibition were also analyzed.

## Figures and Tables

**Table 1:** ^l^H (700MHz) and ^13^C (125MHz) NMR spectroscopic data of compounds **1**–**2** (aspertide A–B) in DMSO-d6.

	1		2	
	*δ*_C_ Type	*δ*_H_ (*J* in Hz)	*δ*_C_ Type	*δ*_H_ (*J* in Hz)
Pro1				
CO	170.8		170.7	
α	57.3	4.29 (dd, *J* = 8.4, 1.9 Hz)	57.7	4.30 (dd, *J* = 8.3, 5.3 Hz)
β	30.7	2.18 m;1.91 m	30.6	2.17 m; 1.92 m
γ	21.9	2.04 m:1.82 m	21.9	2.07 m; 1.85 m
δ	45.9	3.48 m;3.26 m	46.0	3.42 m; 3.37 m
Pro2				
CO	171.3		168.5	
α	51.3	4.37 (dd, *J* = 6.9, 3.8 Hz)	57.5	4.40 (dd, *J* = 8.3, 1.9 Hz,)
β	24.9	1.82 m;1.71 m	28.4	2.09 m; 1.70 m
γ	18.3	1.61 m;1.44 m	25.0	2.02 m; 1.92 m
δ	23.9	1.71 m;1.54 m	46.4	3.44 m; 3.36 m
e	41.8	3.79 m;3.48 m		
Pro3				
CO	170.7		170.8	
α	57.9	4.91 (dd, *J* = 8.5, 5.2 Hz)	58.0	4.84 (dd, *J* = 8.4, 3.7 Hz)
β	29.7	2.34 m;1.68 m	29.7	2.32 m; 1.76 m
γ	22.7	1.82 m;1.71 m	22.7	2.07 m; 1.74 m
δ	46.6	3.39 m	46.3	3.44 m; 3.29 m
Ala				
CO	171.3		171.0	
α	45.8	4.14 (dq, *J* = 8.4, 6.5 Hz)	45.6	4.23 (dt, *J* = 8.6, 6.4 Hz)
β	20.0	1.08 (d, *J* = 6.5 Hz)	20.0	1.09 (d, *J* = 6.5 Hz)
NH		8.16 (d, *J* = 8.5 Hz)		8.25 (d, *J* = 8.5 Hz)
Thr				
CO	167.7		167.8	
α	54.2	4.72 (dd, *J* = 9.2, 1.4 Hz)	54.2	4.72 (dd, *J* = 9.3, 1.4 Hz)
β	73.2	5.00 (qd, *J* = 6.3, 1.4 Hz)	73.0	4.95 (q, *J* = 5.8 Hz)
γ	16.7	1.13 (d, *J* = 6.3 Hz)	16.7	1.11 (d, *J* = 6.3 Hz)
NH		7.71 (d, *J* = 9.2 Hz)		7.85 (d, *J* = 9.3 Hz)
p-Moc				
1	159.5		159.5	
2,6	113.3	6.87 (d, *J* = 8.9 Hz)	113.2	6.87 (d, *J* = 8.9 Hz)
3,5	132.0	7.75 (d, *J* = 8.8 Hz)	132.1	7.75 (d, *J* = 8.8 Hz)
4	127.7		127.8	
7	137.6	6.66 (d, *J* = 13.0 Hz)	137.6	6.66 (d, J = 13.0 Hz)
8	121.5	6.14 (d, *J* = 12.9 Hz)	121.4	6.14 (d, *J* = 12.9 Hz)
9	165.9		165.9	
OCH_3_	55.1	3.76 (s)	55.1	3.76 (s)

## Data Availability

The original data presented in the study are included in the article/Supplementary Materials. Further inquiries can be directed to the corresponding author.

## Supplementary Materials

The following supporting information can be downloaded at: https://www.mdpi.com/article/10.3390/metabo16030159/s1, Figures S1–S31: The NMR, HRESIMS, UV, and IR spectra of **1**−**5**; ITS sequence data of the strain; bioassay data for acetylcholinesterase and neuraminidase inhibition.
