# Supplementary material for: Two New Pentadepsipeptides from the Mangrove Fungus Aspergillus sp. SCSIO 41443
Source: Metabolites. 2026 Feb 27;16(3):159. doi: 10.3390/metabo16030159 (PMC13027552; doi:10.3390/metabo16030159)
Supplement: Supplementary file 1 [file metabolites-16-00159-s001.zip › metabolites-4119851-supplementary.pdf]

Two New Pentadepsipeptides from the Mangrove Fungus *Aspergillus* sp. SCSIO 41443

Ying Liu [1,2], Yi Chen [2,3], Jiao Xiao [1,*], Xin Sun [2,4], Xuefeng Zhou [2,3], Yonghong Liu [1,2,3] and Bin Yang [2,3,*]

1 Wuya College of Innovation, Shenyang Pharmaceutical University, Shenyang 110016, China; 15248089565@163.com (Y.L.); yonghongliu@scsio.ac.cn (Y.L.)
2 State Key Laboratory of Tropical Oceanography/Guangdong Key Laboratory of Marine Materia Medica, South China Sea Institute of Oceanology, Chinese Academy of Sciences, Guangzhou 510301, China; chenyi221@mails.ucas.ac.cn (Y.C.); 18844971326@163.com (X.S.); xfzhou@scsio.ac.cn (X.Z.)
3 University of Chinese Academy of Sciences, Beijing 100049, China
4 Shenzhen Clinical College of Integrated Chinese and Western Medicine, Guangzhou University of Chinese Medicine, Shenzhen 518104, China
* Correspondence: xj110121@126.com (J.X.); yangbin@scsio.ac.cn (B.Y.)

Abstract

Background: Mangrove fungi are a prolific source of structurally diverse natural products. Among these, natural peptides with varied biological activities hold high commercial value and have been successfully developed into drugs for treating numerous diseases. **Methods**: Following rice solid-state fermentation of the strain, extracellular metabolites were extracted from the culture filtrate to obtain a crude extract. Cyclic depsipeptides were isolated from the crude extract by silica gel vacuum liquid chromatography for preliminary fractionation and enrichment, followed by high-performance liquid chromatography (HPLC) purification. The structures of the compounds were determined based on extensive spectroscopic analysis (1D and 2D NMR, ESI-MS-MS analysis) and Marfey's method for amino acid configuration assignment. **Results**: Ultimately, two new compounds, aspertides F (**1**) and G (**2**), and three known compounds, aspertides C, D, and A (**3**–**5**), were identified. The bioassays indicated that these compounds exhibited weak activity against acetylcholinesterase and neuraminidase. **Conclusions:** The research findings on this strain have not only enriched the metabolic resource library of mangrove fungi but also highlighted their diverse biological activities and significant application potential.

Keywords: mangroves; *Aspergillus* sp.; pentadepsipeptides; structures

Figure S1: ^{1}H NMR spectrum of 1 in DMSO-*d6*. 4

Figure S2: ^{13}C NMR spectrum of 1 in DMSO-*d6*. 4

Figure S3: HSQC spectrum of 1 in DMSO-*d6*. 5

Figure S4: HMBC spectrum of 1 in DMSO-*d6*. 5

Figure S5: ^{1}H-^{1}H COSY spectrum of 1 in DMSO-*d6*. 6

Figure S6: NOESY spectrum of 1 in DMSO-*d6*. 6

Figure S7: HR-ESI-MS spectrum of 1. 7

Figure S8: ESI-MS/MS spectrum of 1. 7

Figure S9: IR spectrum of 1. 8

Figure S10: UV spectrum of 1 in MeOH. 9

Figure S11: CD spectrum of 1 in MeOH. 9

Figure S12: ^{1}H NMR spectrum of 2 in DMSO-*d6*. 10

Figure S13: ^{13}C NMR spectrum of 2 in DMSO-*d6*. 10

Figure S14: HSQC spectrum of 2 in DMSO-*d6*. 11

Figure S15: HMBC spectrum of 2 in DMSO-*d6*. 11

Figure S16: ^{1}H-^{1}H COSY spectrum of 2 in DMSO-*d6*. 12

Figure S17: NOESY spectrum of 2 in DMSO-*d6*. 12

Figure S18: HR-ESI-MS spectrum of 2. 13

Figure S19: IR spectrum of 2. 13

Figure S20: UV spectrum of 2 in MeOH. 14

Figure S21: CD spectrum of 2 in MeOH. 14

Figure S22: HPLC analysis of FDAA derivatives of standard amino acids (columns： NanoChrom ChromCore ODS, 120 C18, 10 × 250 mm, 5 μm). 15

Figure S23: HPLC analysis of FDAA derivatives of compounds 1−2 (columns: NanoChrom ChromCore ODS, 120 C18, 10 × 250 mm, 5 μm). 18

Figure S24: ^{1}H NMR spectrum of 3 in DMSO-*d*6. 19

Figure S25: ^{13}C NMR spectrum of 3 in DMSO-*d6*. 19

Figure S26: ^{1}H NMR spectrum of 4 in DMSO-*d6*. 20

Figure S27: ^{13}C NMR spectrum of 4 in DMSO-*d6*. 20

Figure S28: ^{1}H NMR spectrum of 5 in DMSO-*d6*. 21

Figure S29: ^{13}C NMR spectrum of 5 in DMSO-*d6*. 21

Figure S30: Acetylcholinesterase Inhibition Data for the Positive Control and Compounds 1–5. ... 22

Figure S31: Neuraminidase Inhibition Data and Standard Curve for the Compounds 1–5. ... 22

Figure S1: ^{1}H NMR spectrum of 1 in DMSO-*d6*.

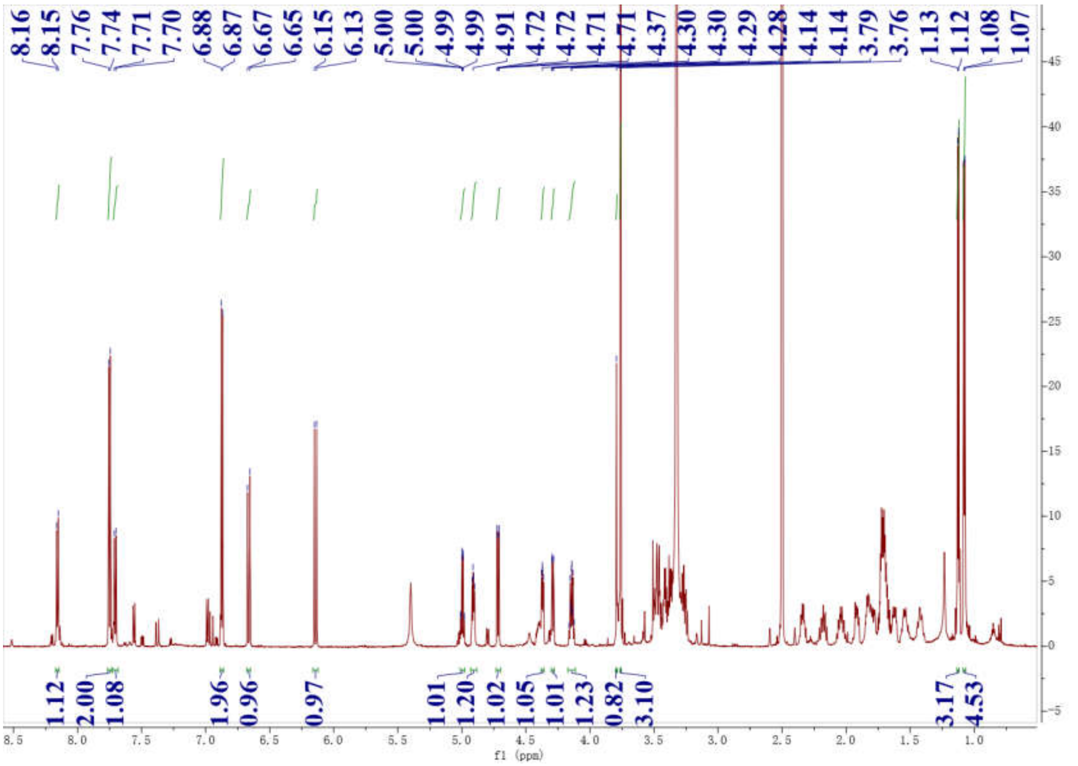

Figure S2: ^{13}C NMR spectrum of **1** in DMSO-*d6*.

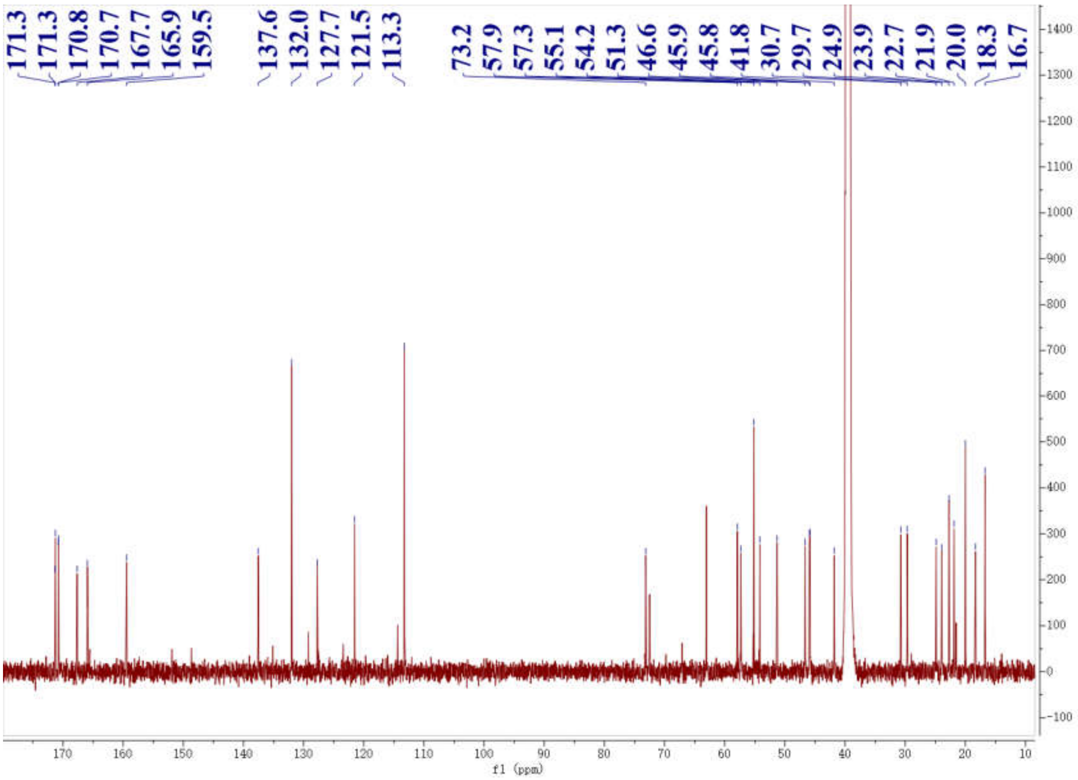

Figure S3: HSQC spectrum of **1** in DMSO-*d6*.

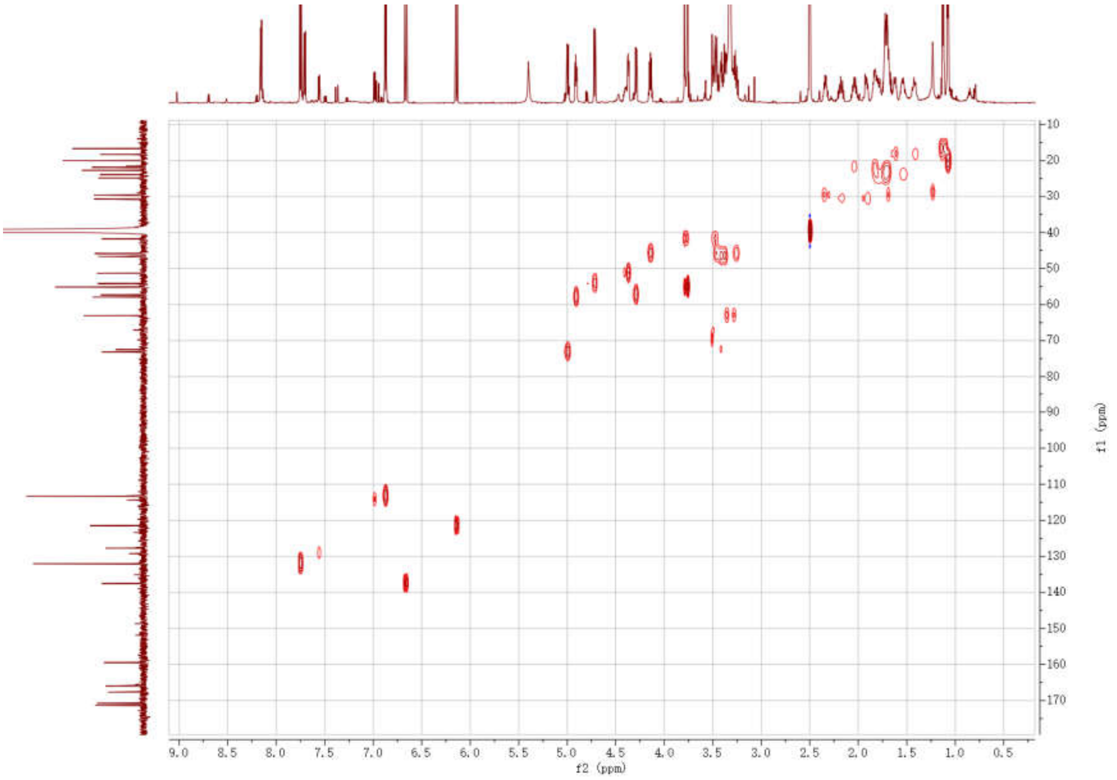

Figure S4: HMBC spectrum of **1** in DMSO-*d6*.

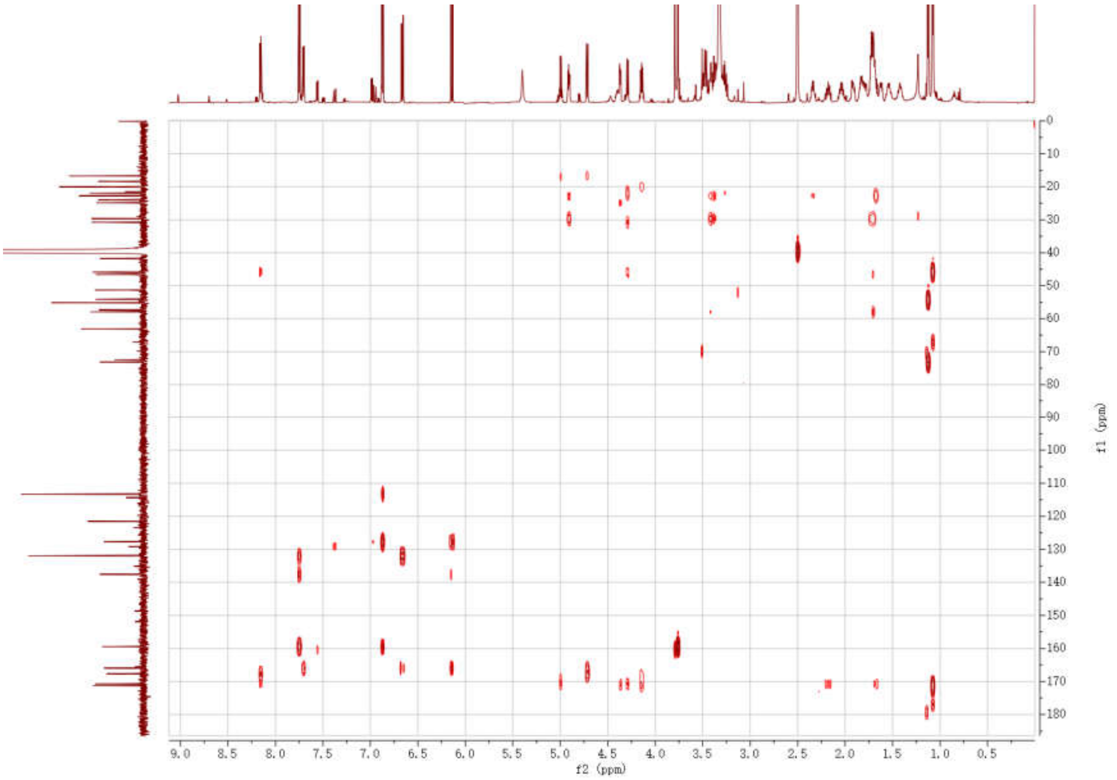

Figure S5: ^{1}H-^{1}H COSY spectrum of **1** in DMSO-*d6*.

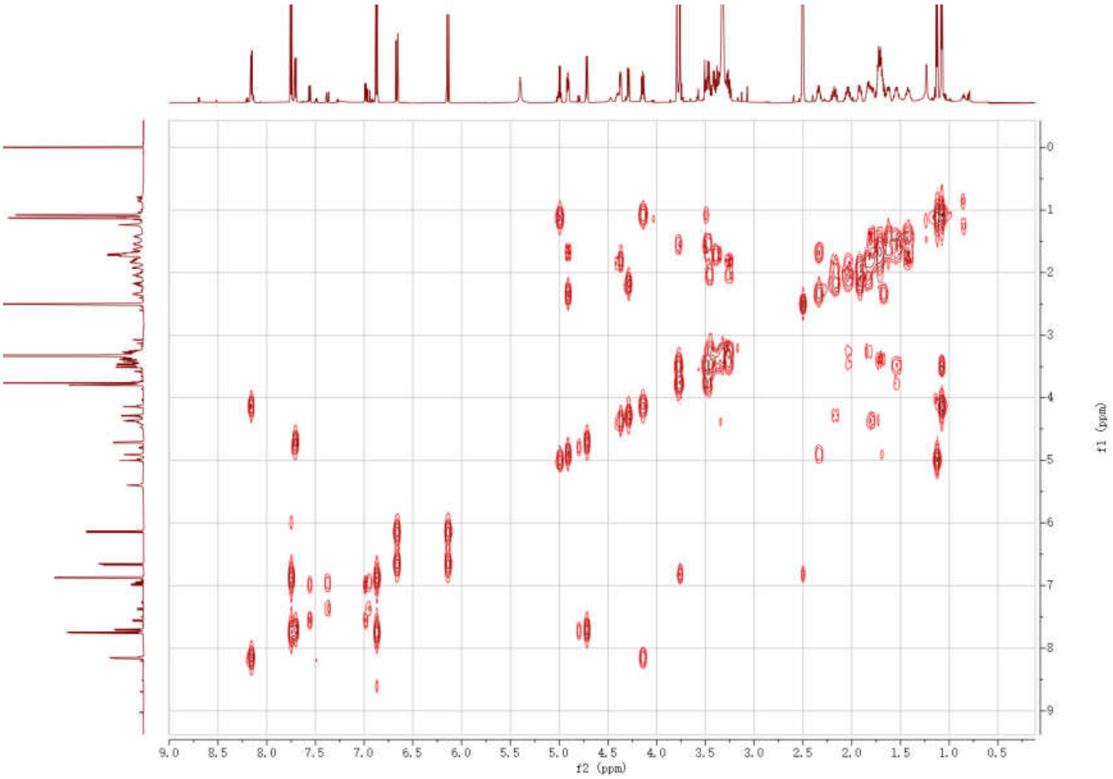

Figure S6: NOESY spectrum of **1** in DMSO-*d6*.

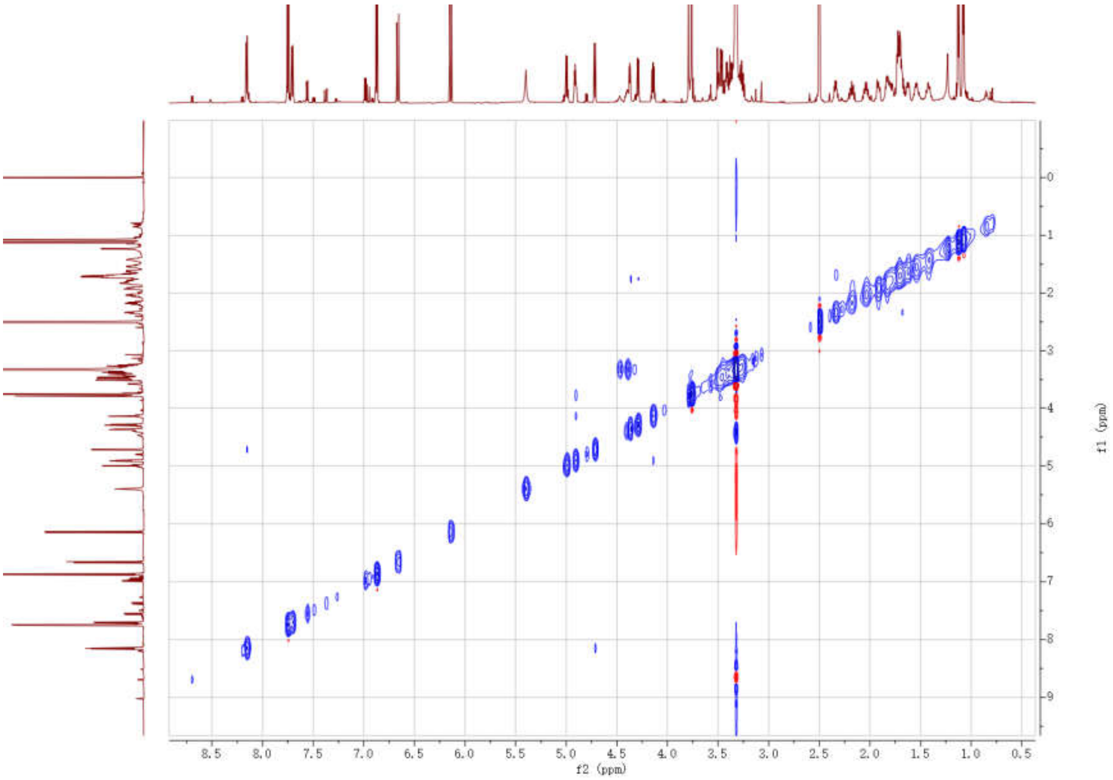

Figure S7: HR-ESI-MS spectrum of **1**.

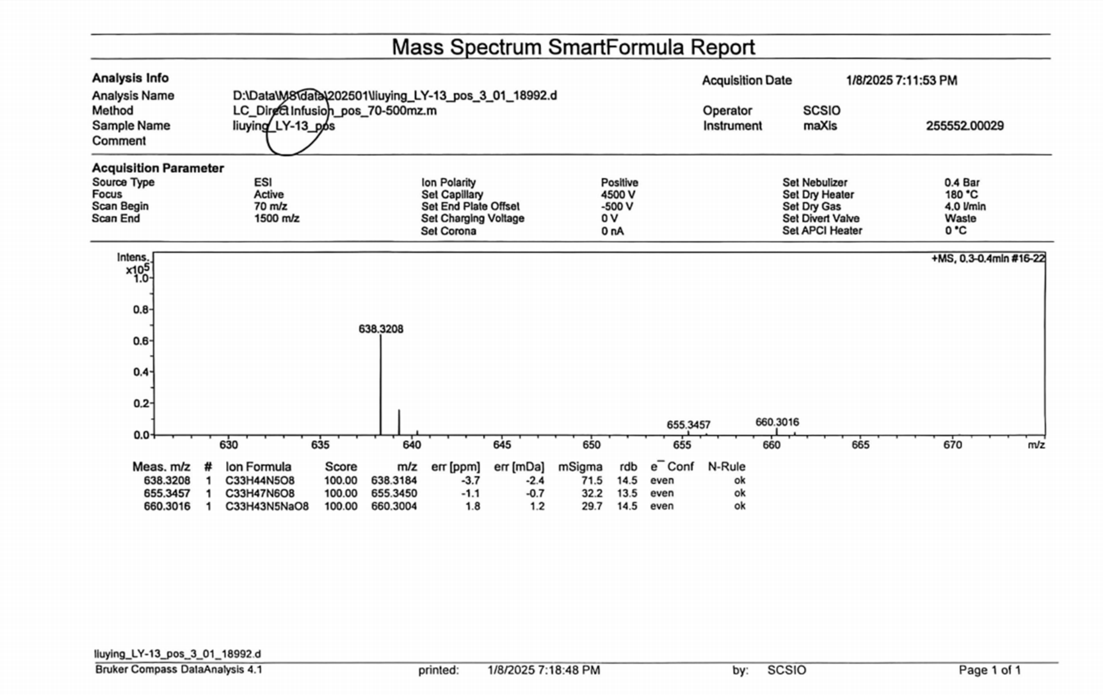

Figure S8: ESI-MS/MS spectrum of **1**.

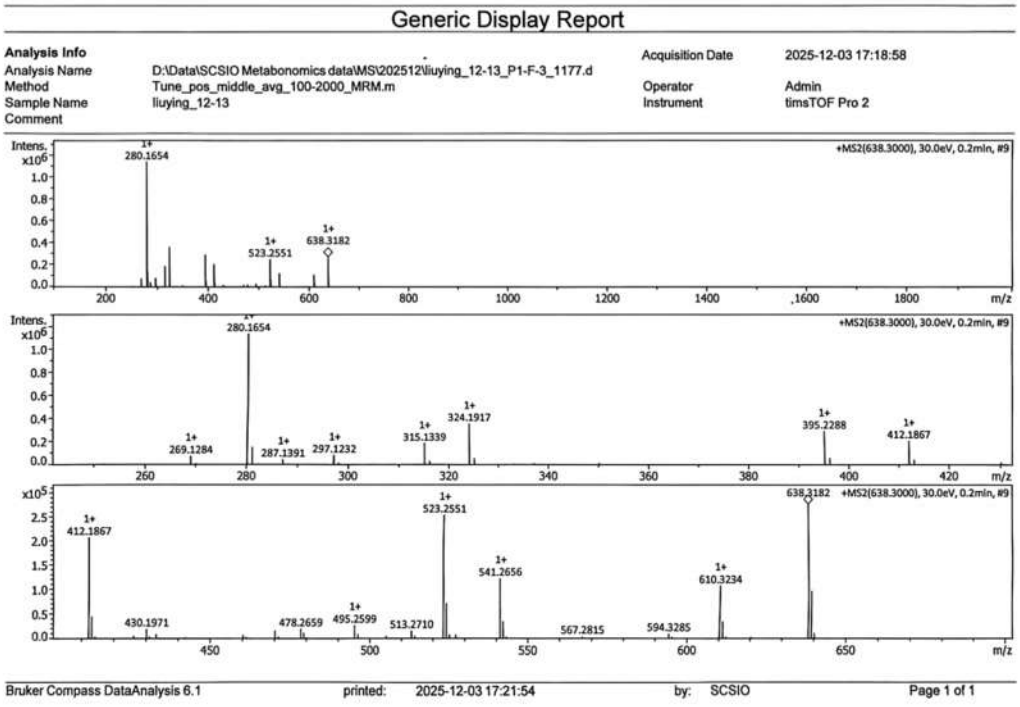

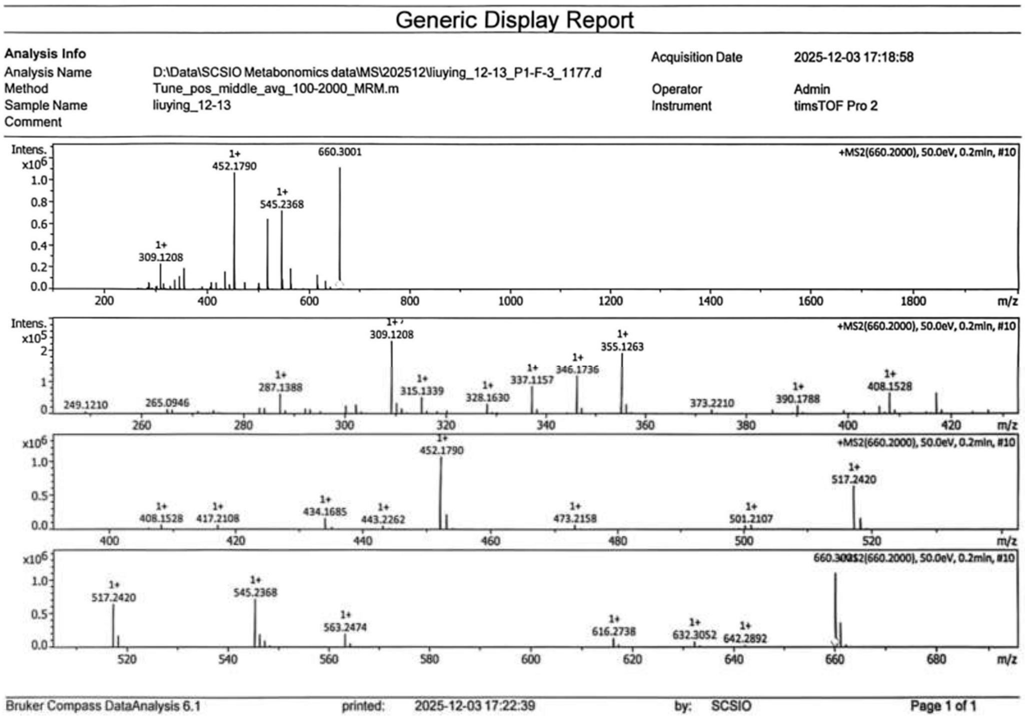

Figure S9: IR spectrum of **1**.

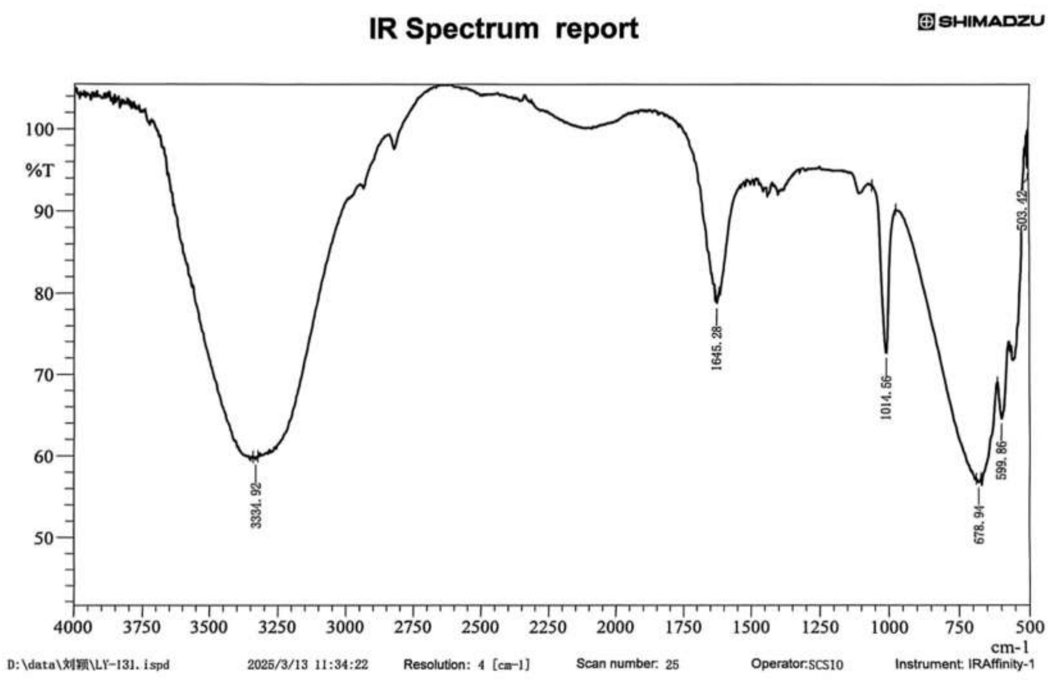

Figure S10: UV spectrum of **1** in MeOH.

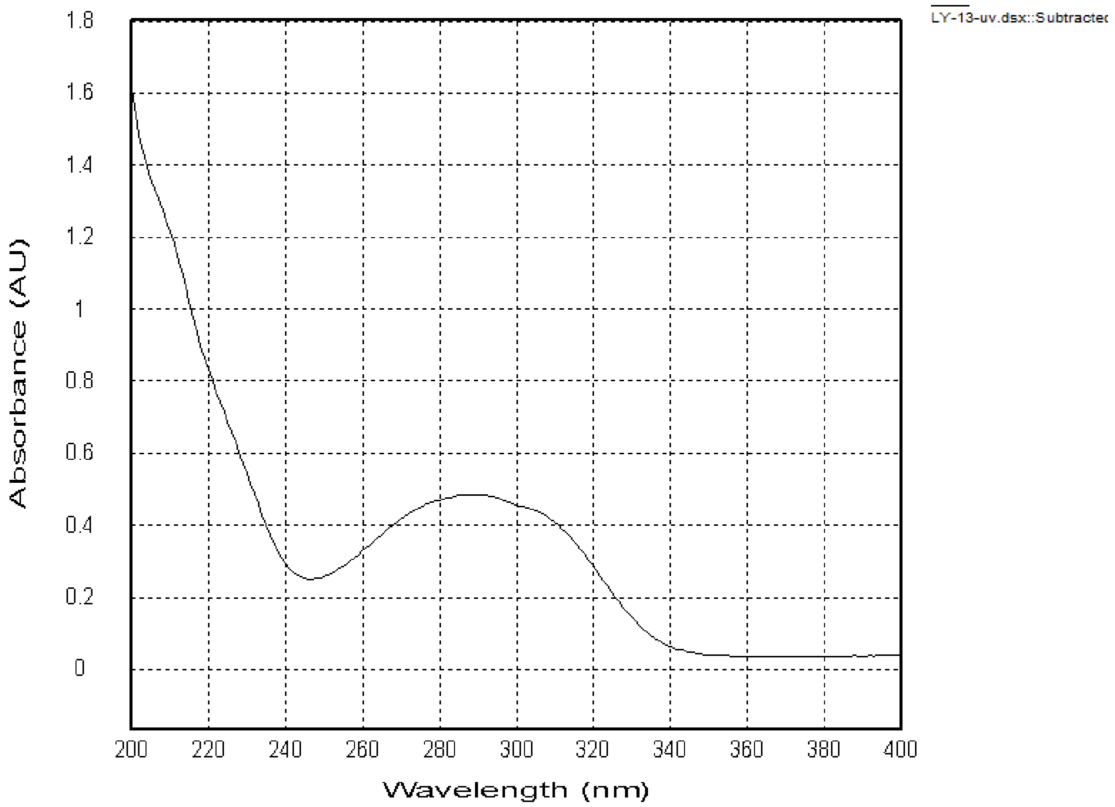

Figure S11: CD spectrum of **1** in MeOH.

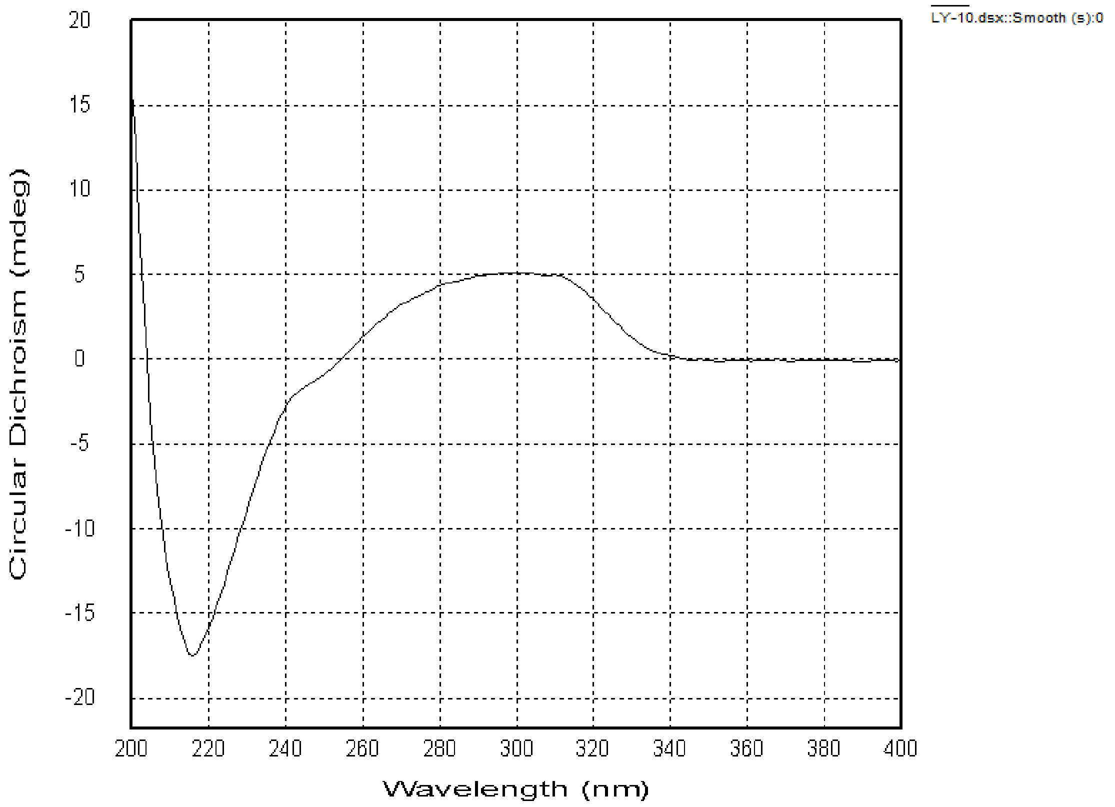

Figure S12: ^{1}H NMR spectrum of **2** in DMSO-*d6*.

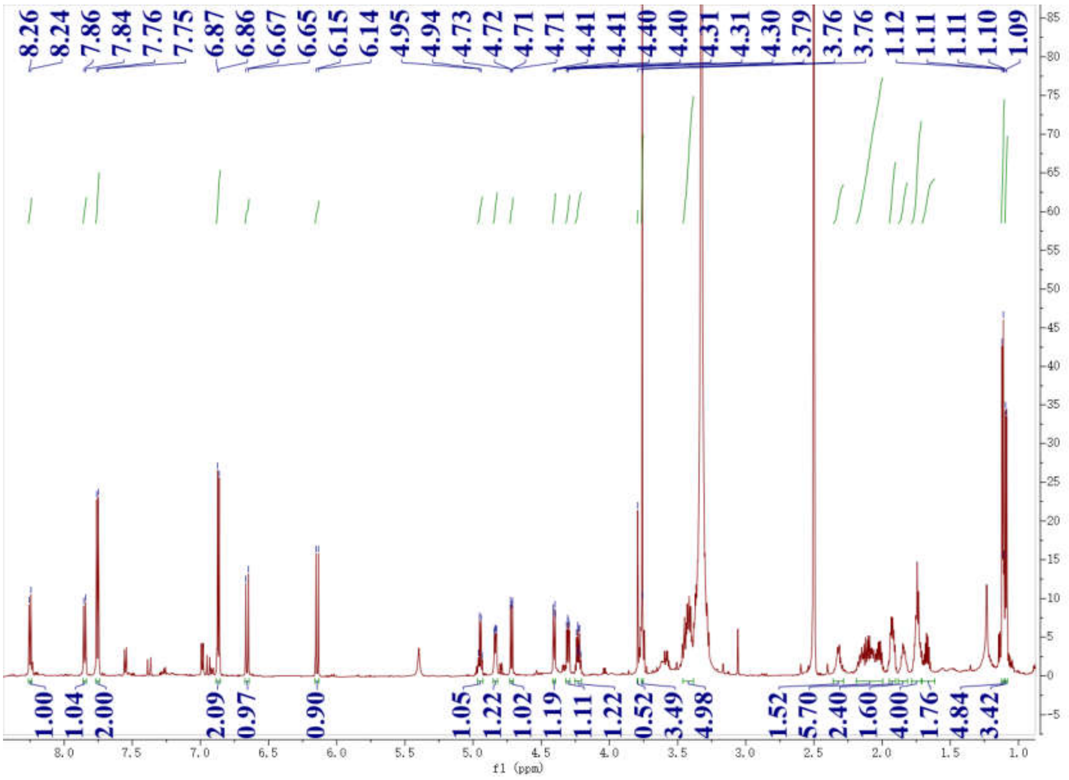

Figure S13: ^{13}C NMR spectrum of **2** in DMSO-*d6*.

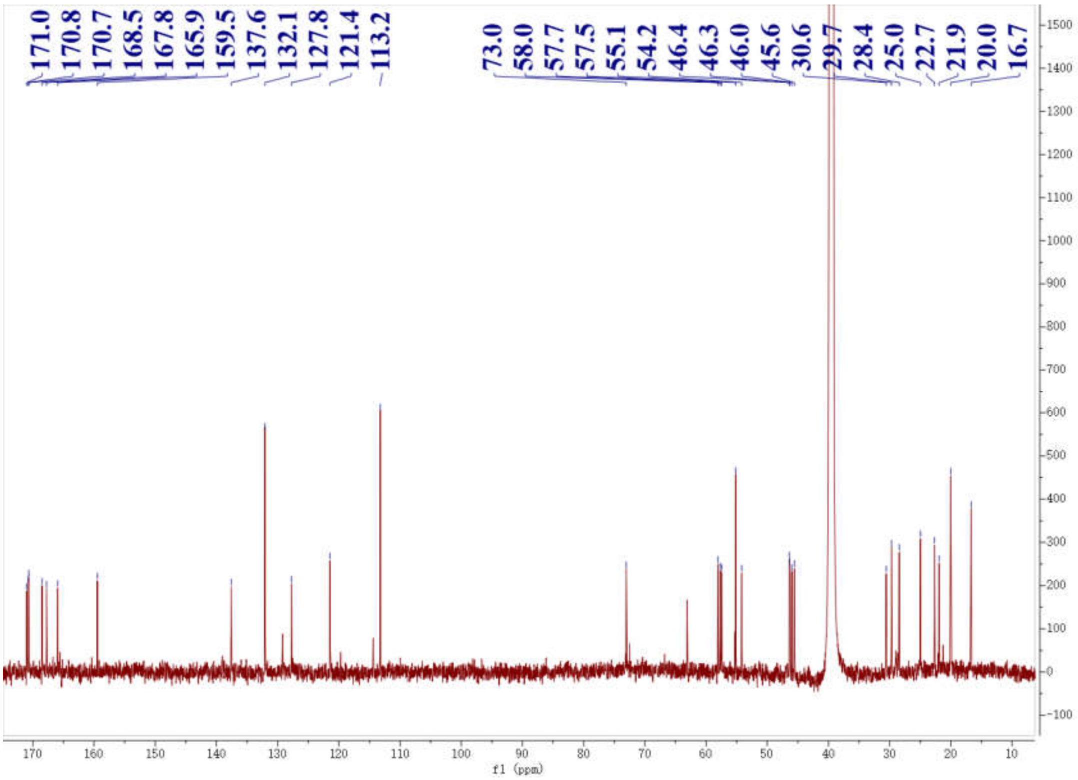

Figure S14: HSQC spectrum of **2** in DMSO-*d6*.

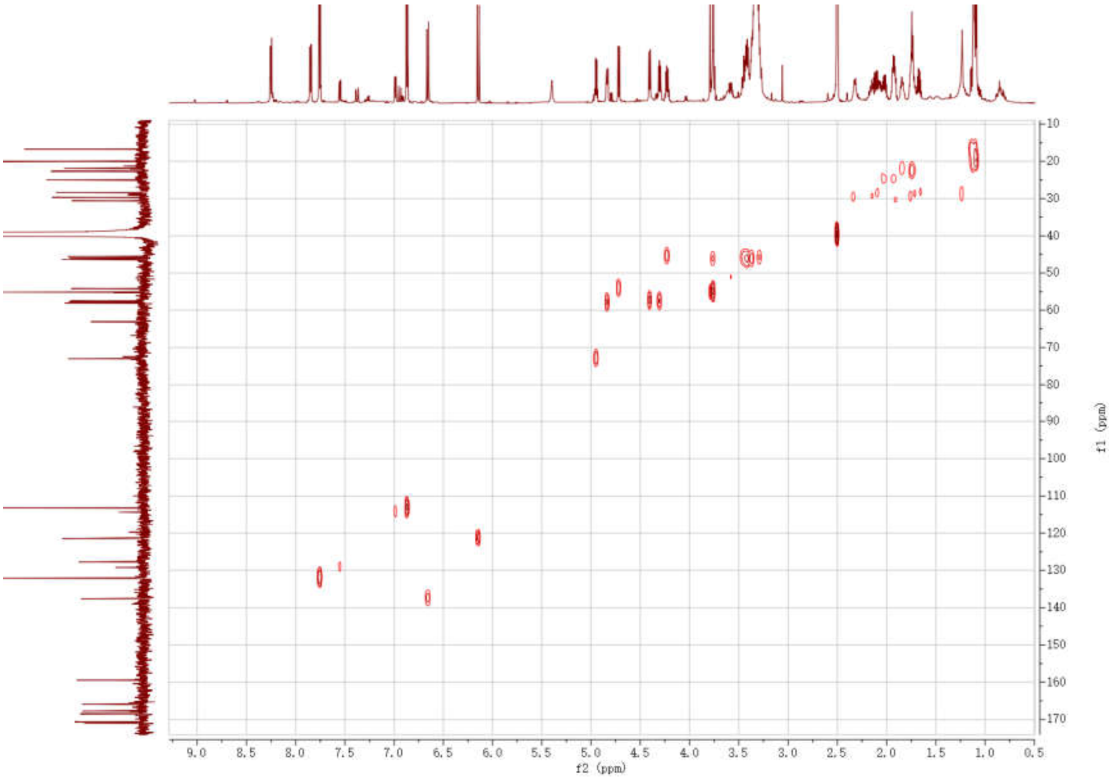

Figure S15: HMBC spectrum of **2** in DMSO-*d6*.

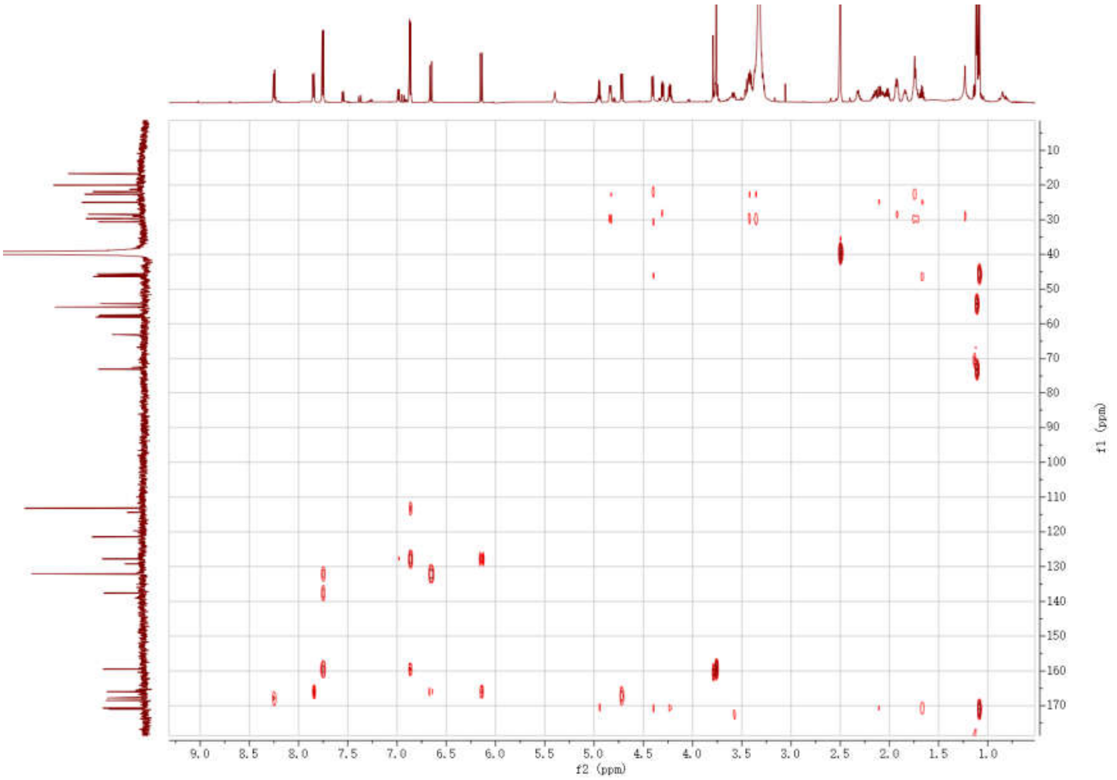

Figure S16: ^{1}H-^{1}H COSY spectrum of **2** in DMSO-*d6*.

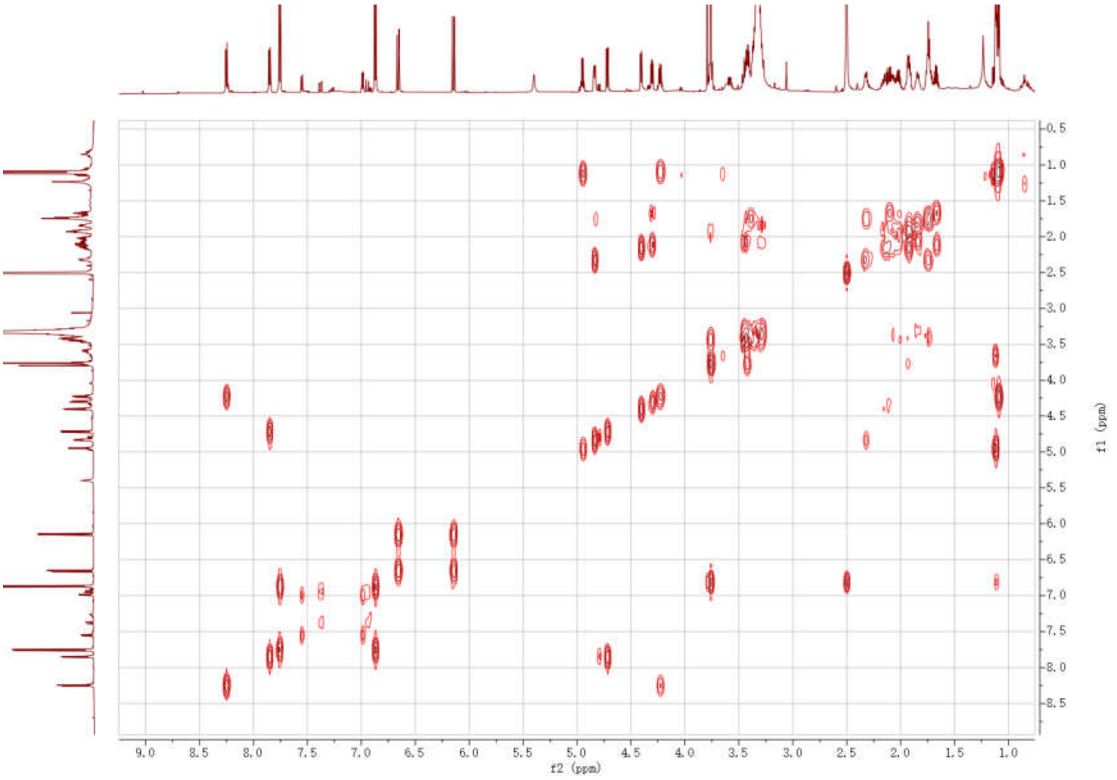

Figure S17: NOESY spectrum of **2** in DMSO-*d6*.

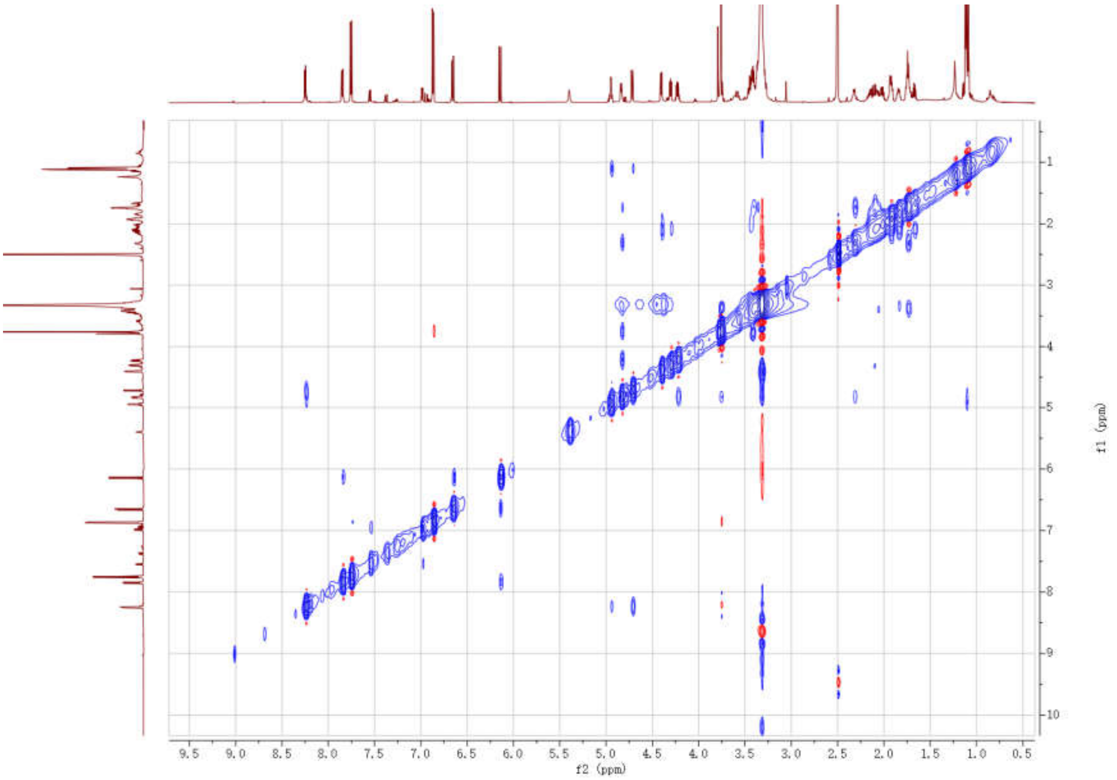

Figure S18: HR-ESI-MS spectrum of **2**.

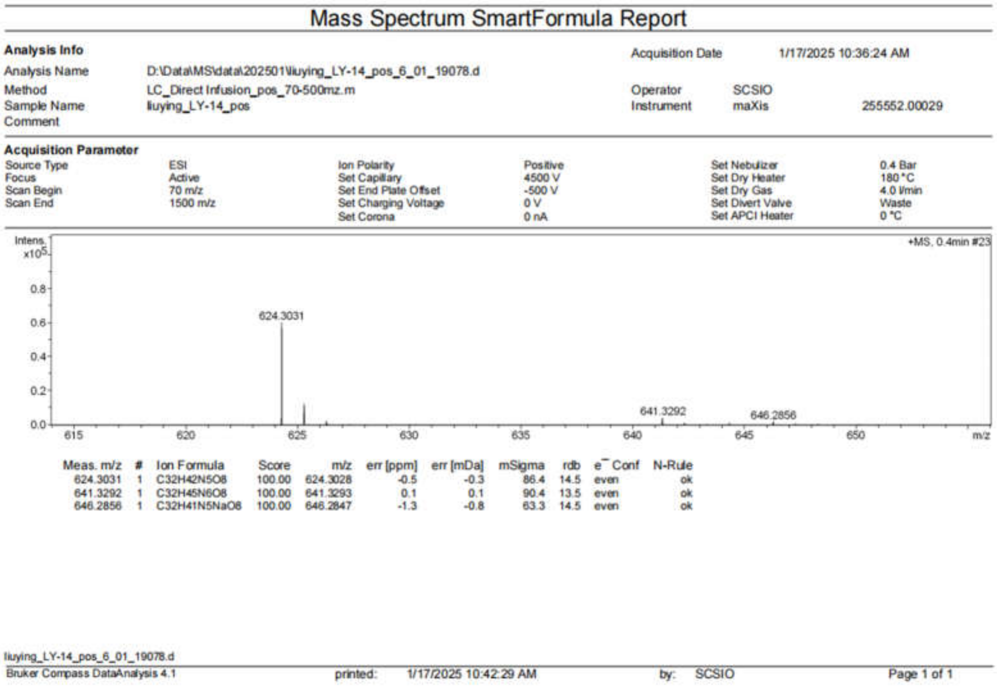

Figure S19: IR spectrum of **2**.

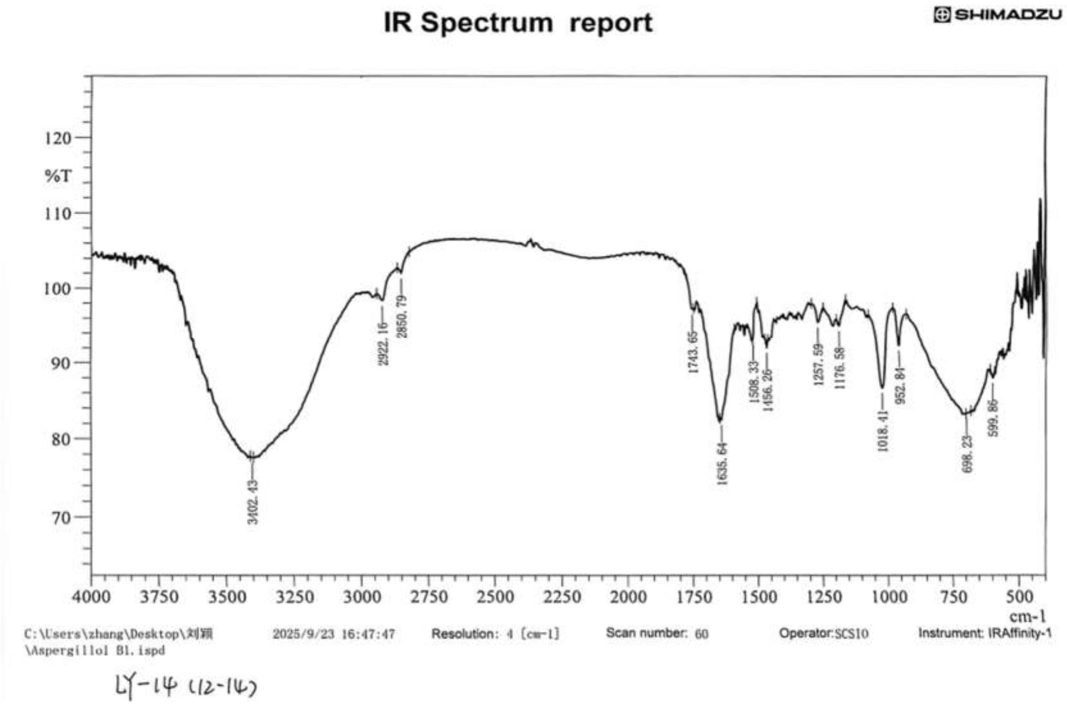

Figure S20: UV spectrum of **2** in MeOH.

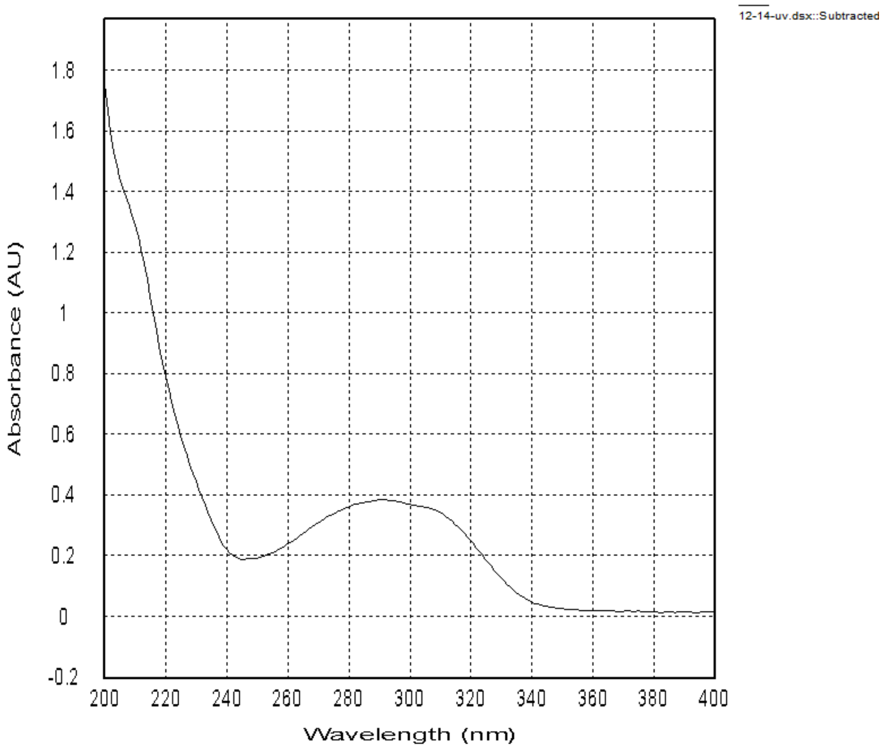

Figure S21: CD spectrum of **2** in MeOH.

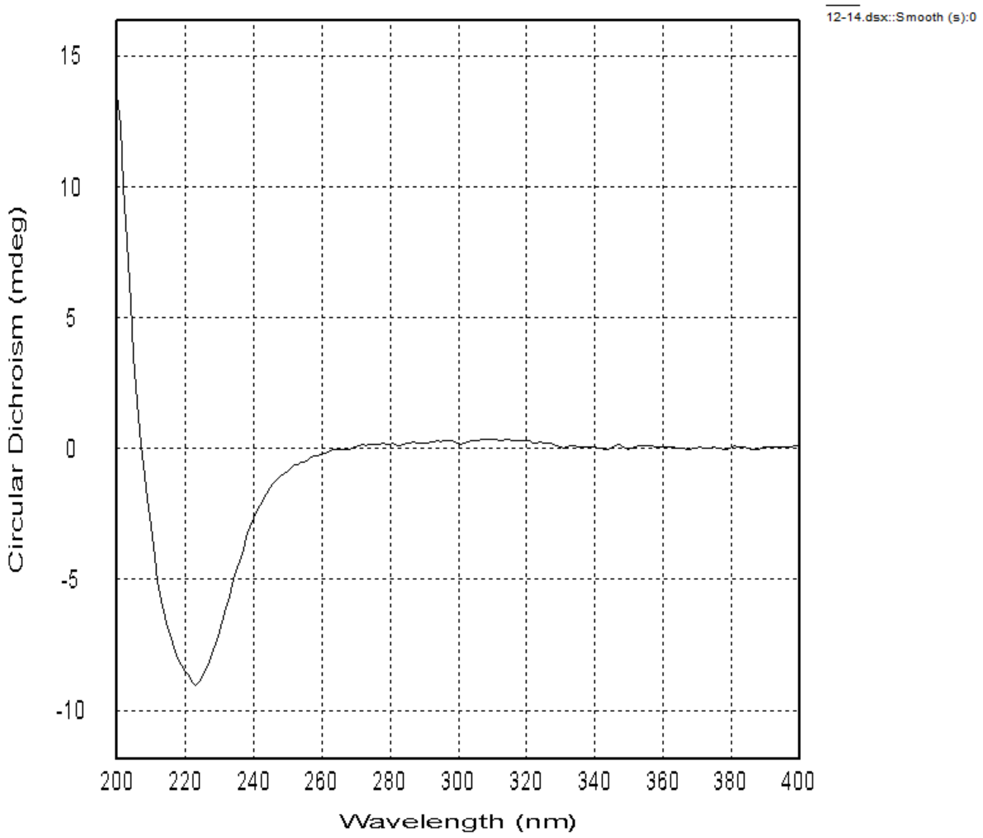

Figure S22: HPLC analysis of FDAA derivatives of standard amino acids (columns：NanoChrom ChromCore ODS, 120 C18, 10 × 250 mm, 5 μm).

FDAA

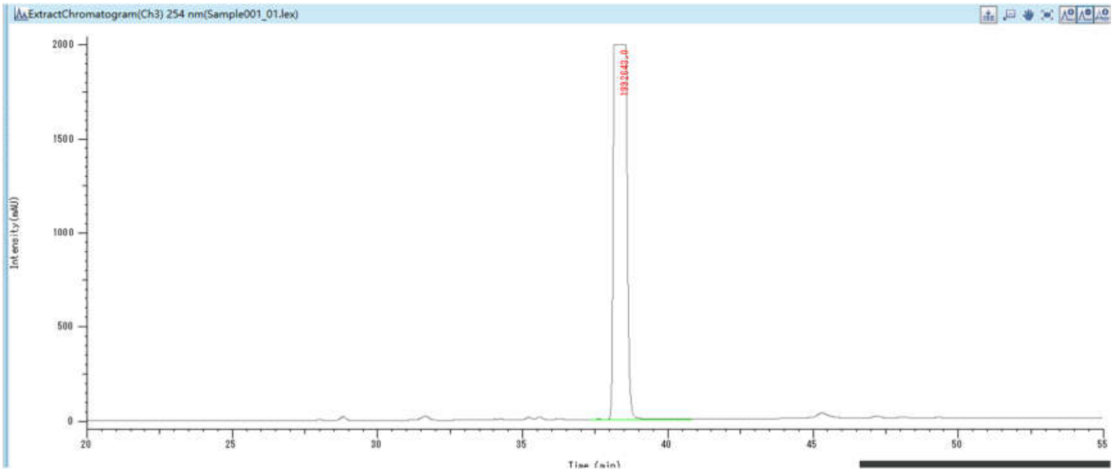

L-Thr−FDAA

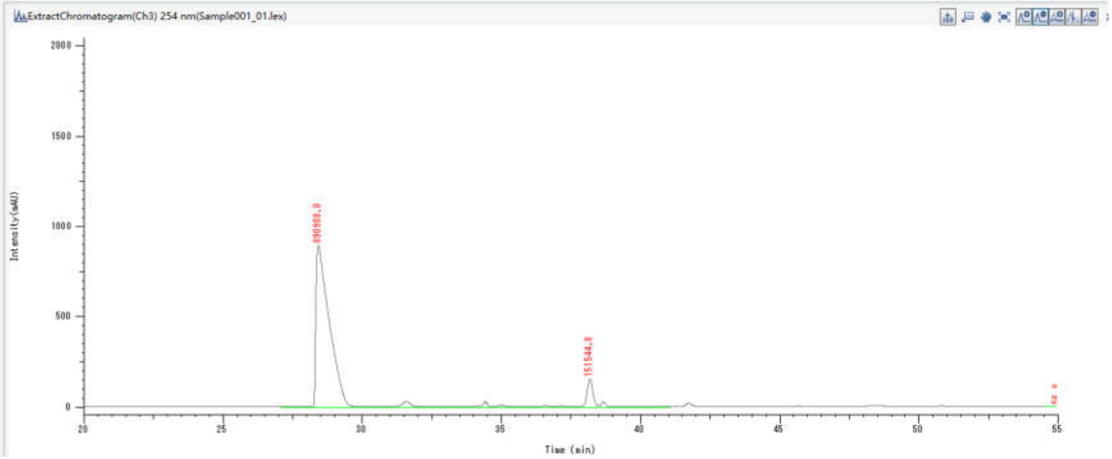

D-Thr−FDAA

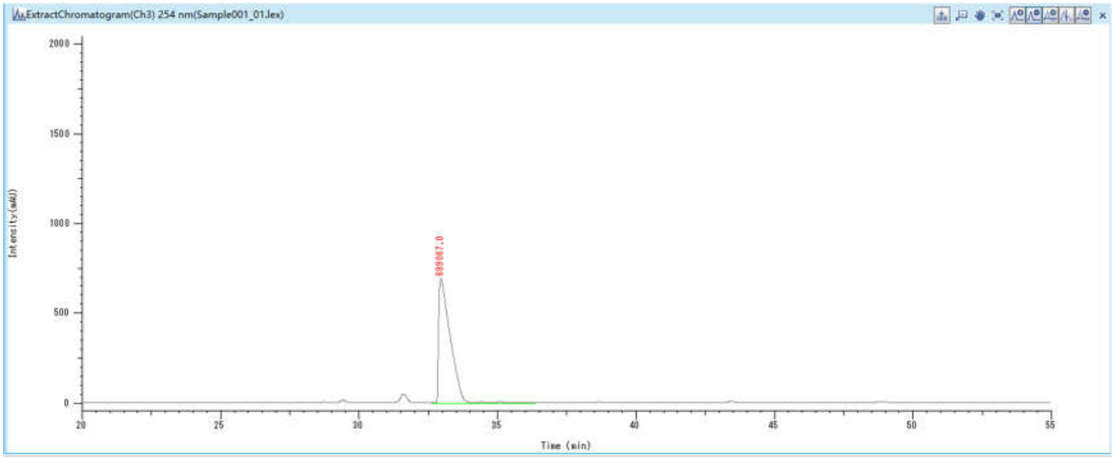

L-Ala−FDAA

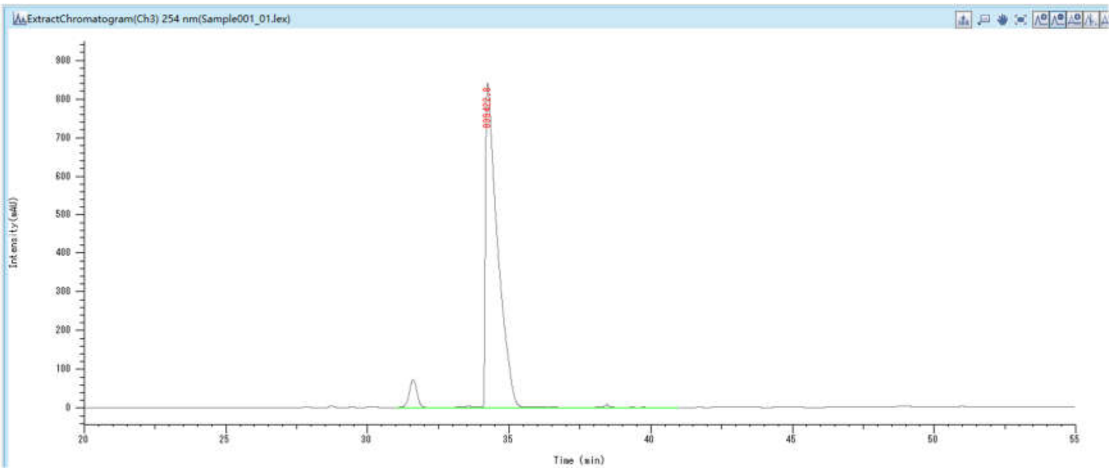

D-Ala−FDAA

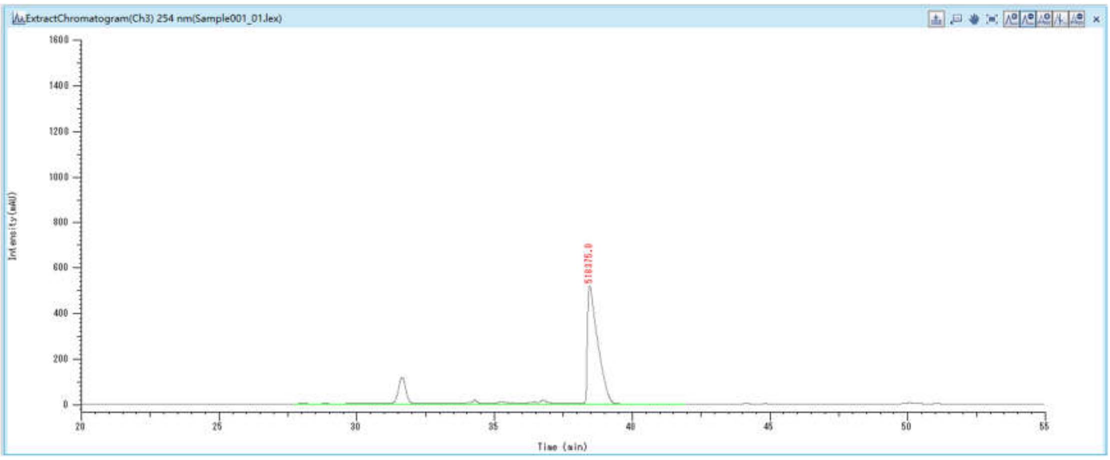

L-Pro−FDAA

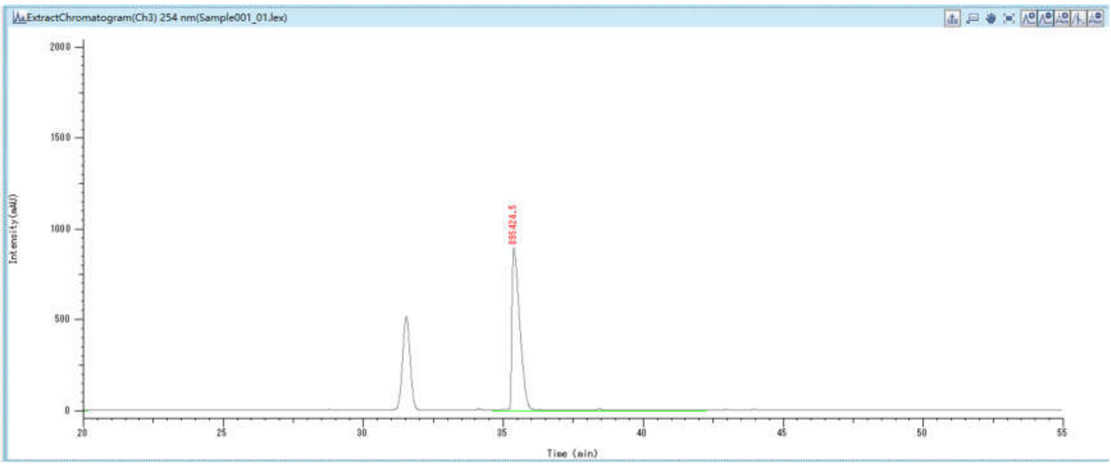

D-Pro−FDAA

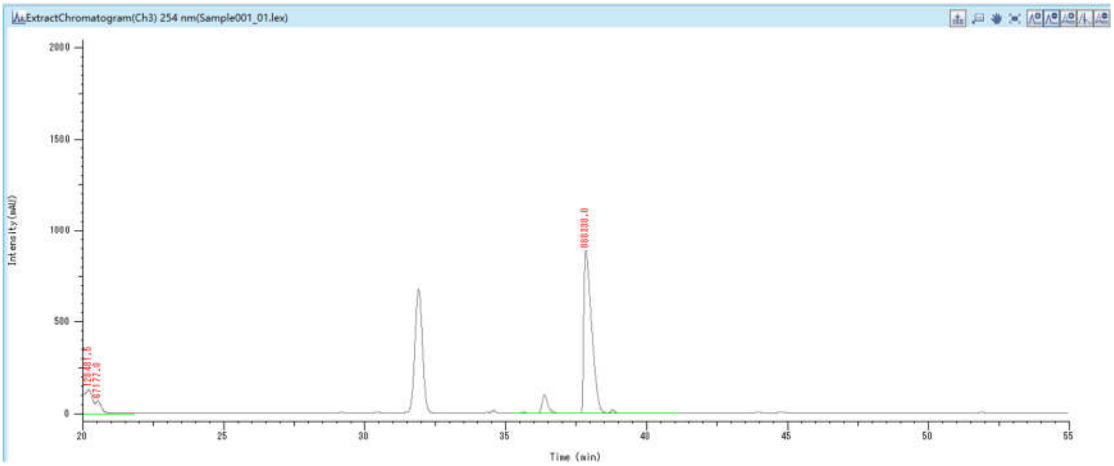

L-Pip−FDAA

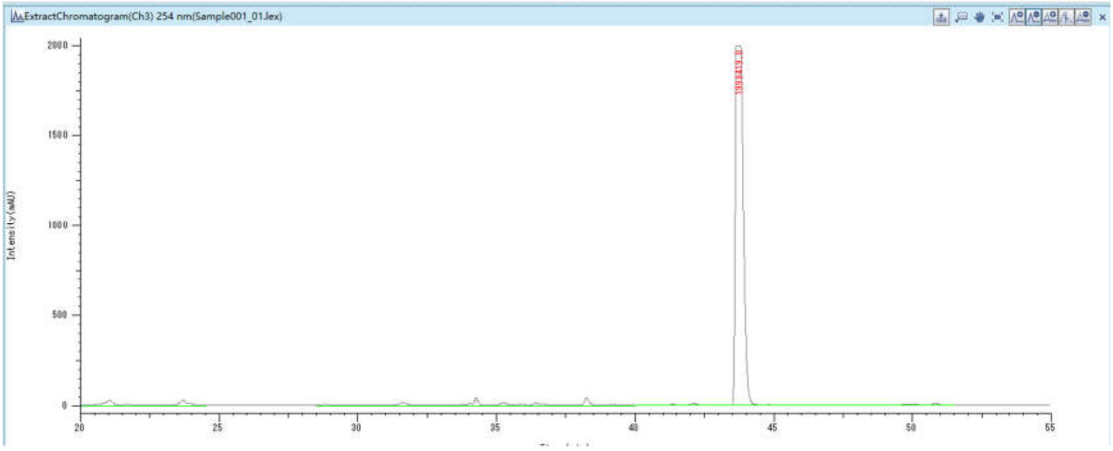

D-Pip−FDAA

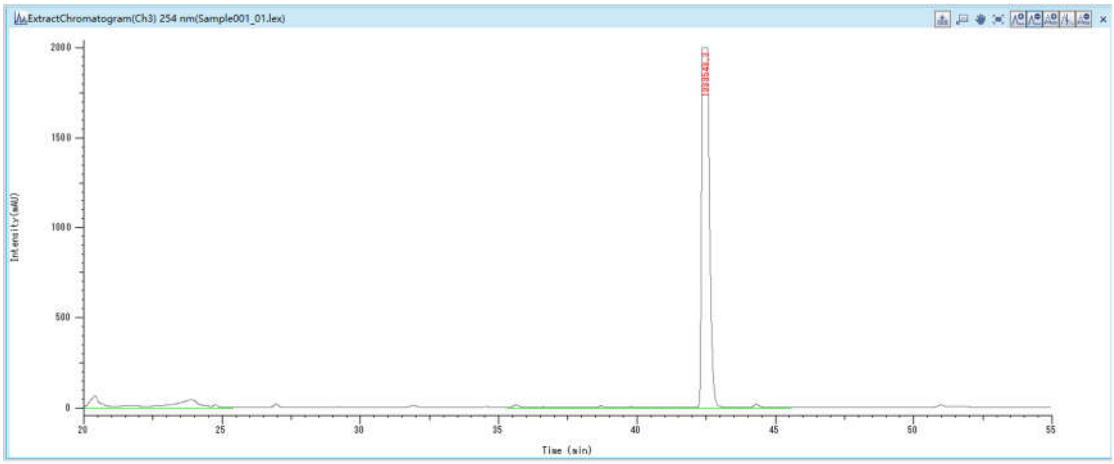

Figure S23: HPLC analysis of FDAA derivatives of compounds **1**−**2** (columns: NanoChrom ChromCore ODS, 120 C18, 10 × 250 mm, 5 μm).

Compound **1**−FDAA

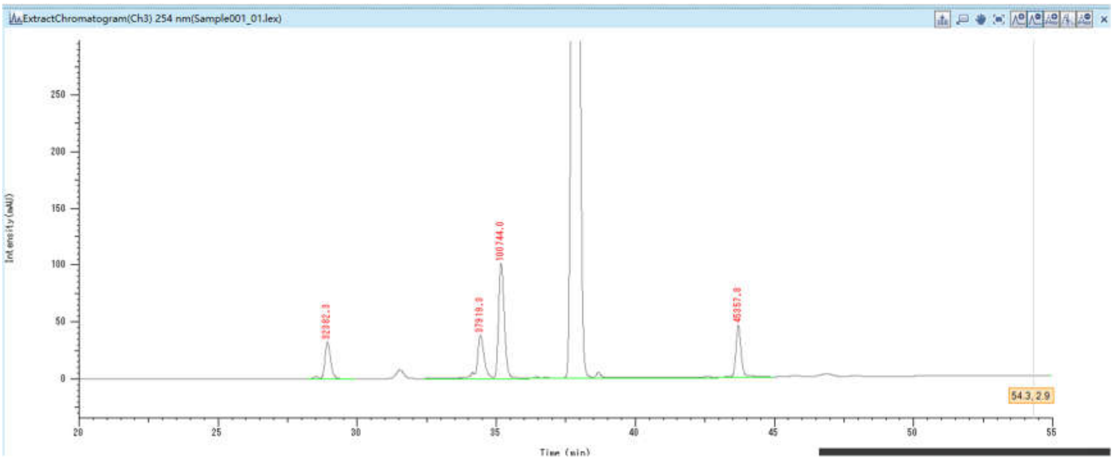

Compound **2**−FDAA

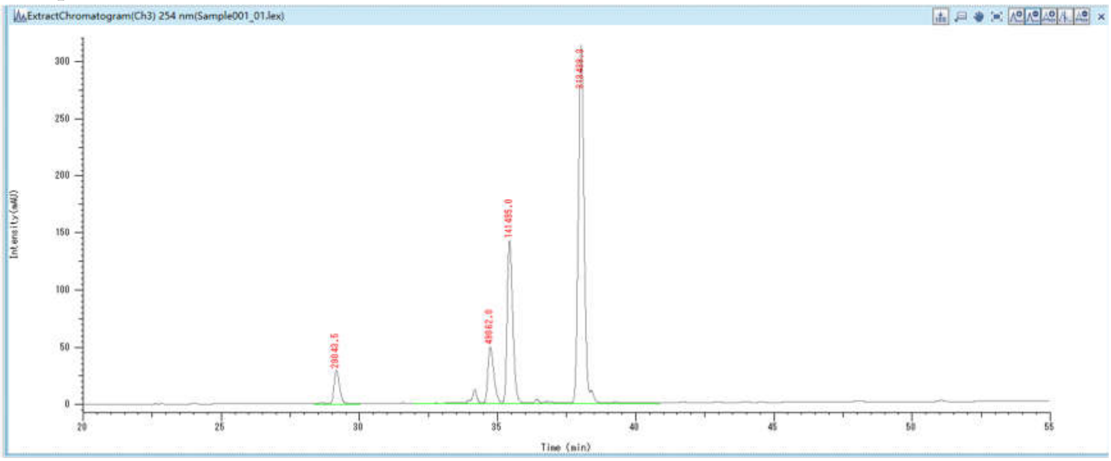

Figure S24: ^{1}H NMR spectrum of **3** in DMSO-*d6*.

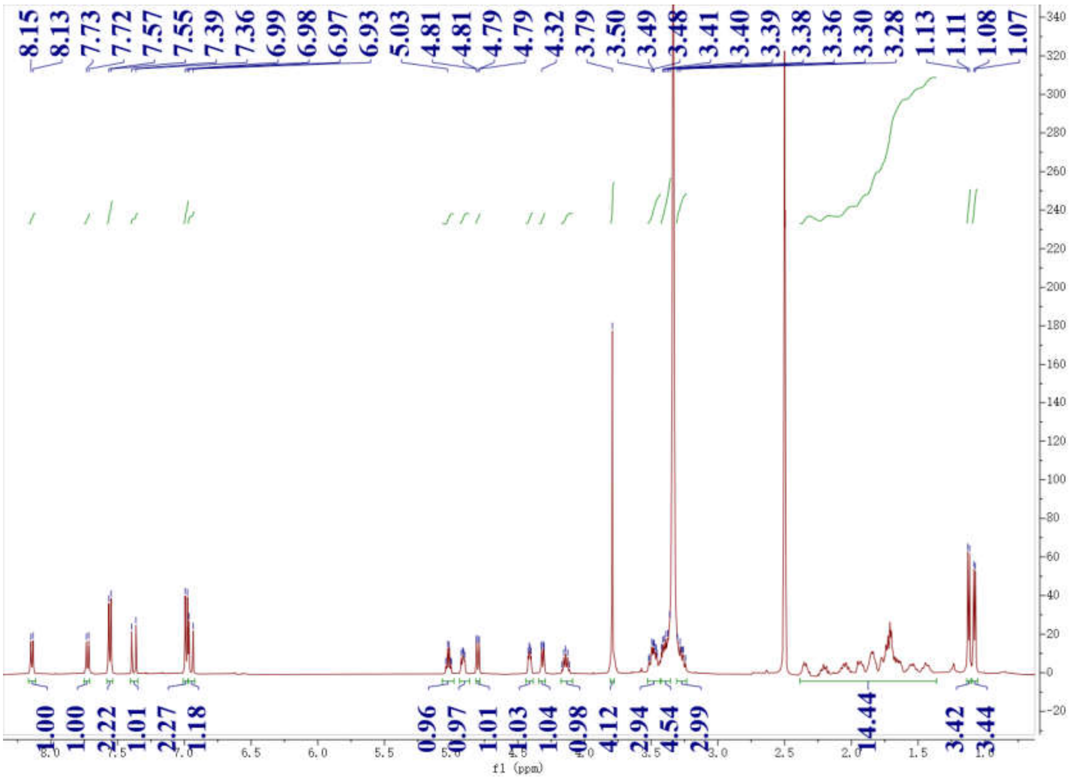

Figure S25: ^{13}C NMR spectrum of **3** in DMSO-*d6*.

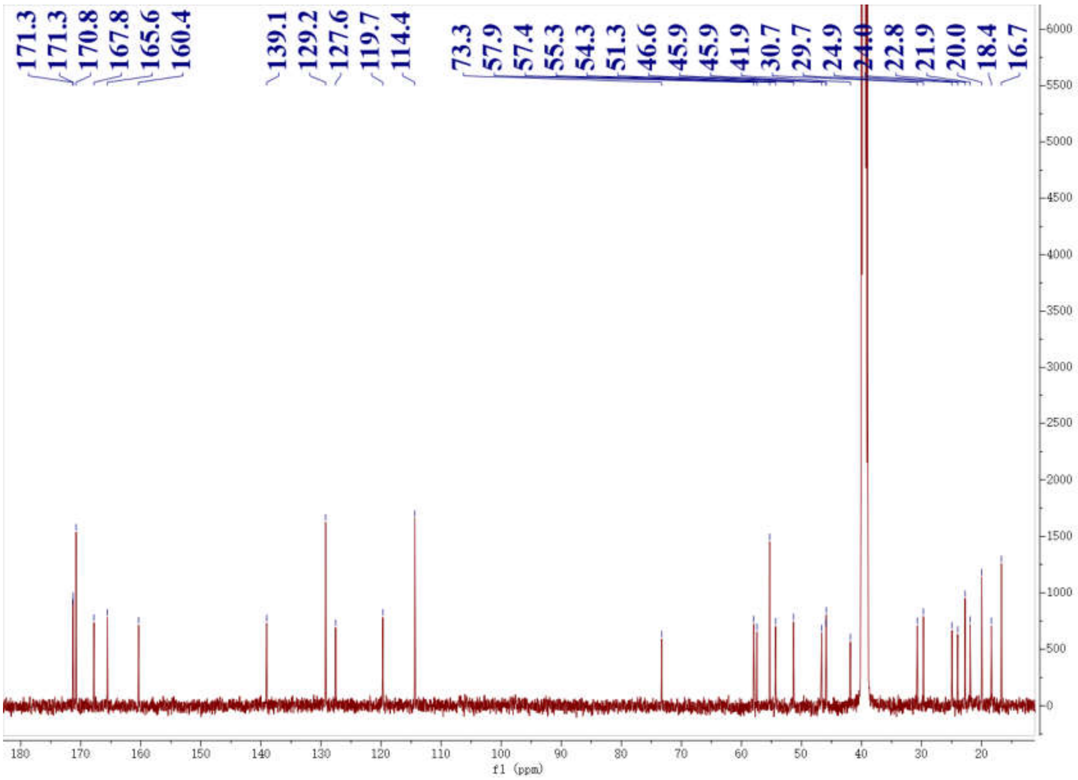

Figure S26: ^{1}H NMR spectrum of **4** in DMSO-*d6*.

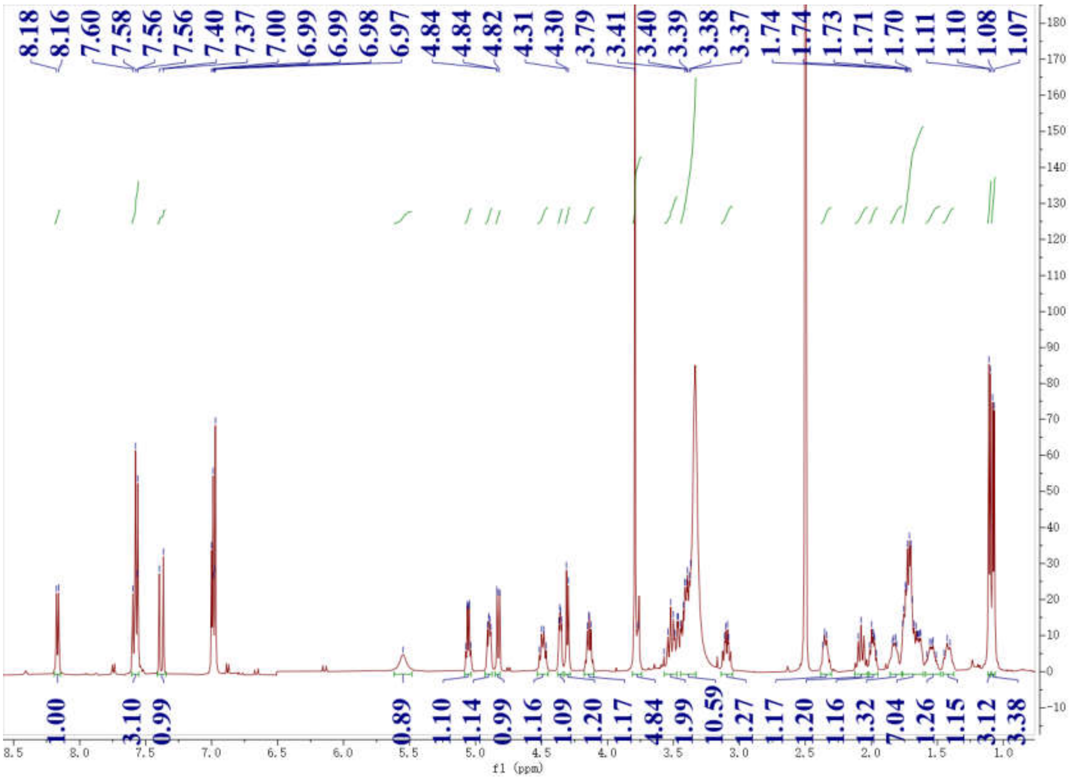

Figure S27: ^{13}C NMR spectrum of **4** in DMSO-*d6*.

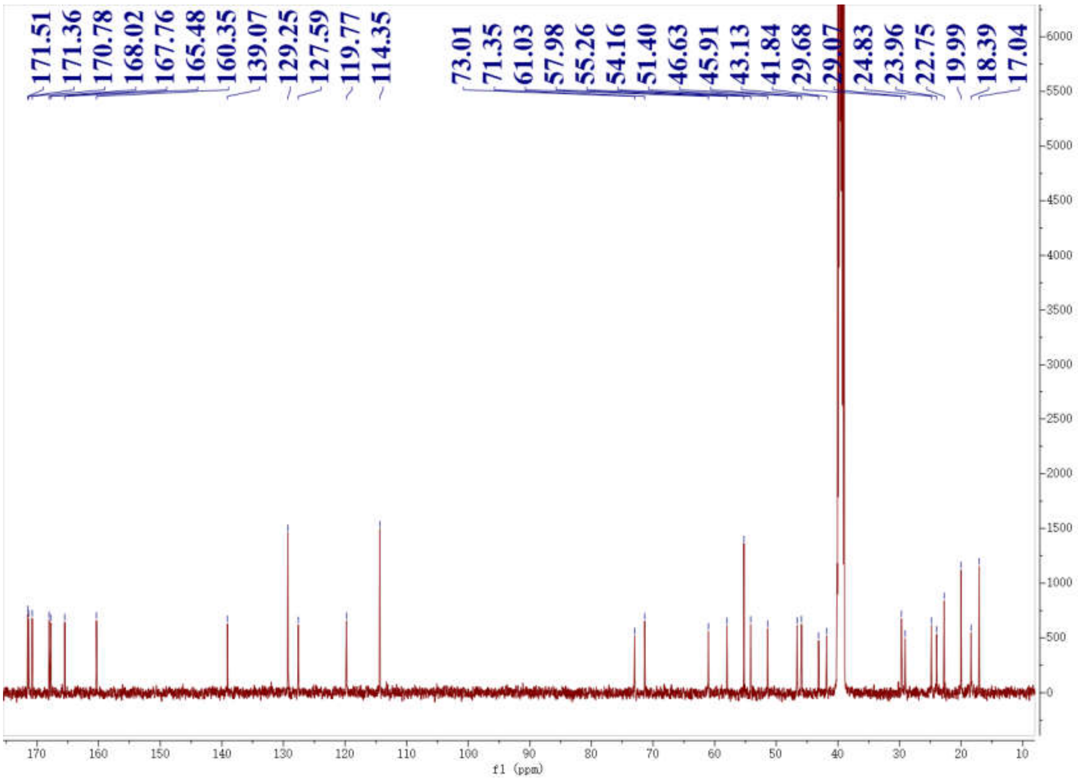

Figure S28: ^{1}H NMR spectrum of **5** in DMSO-*d6*.

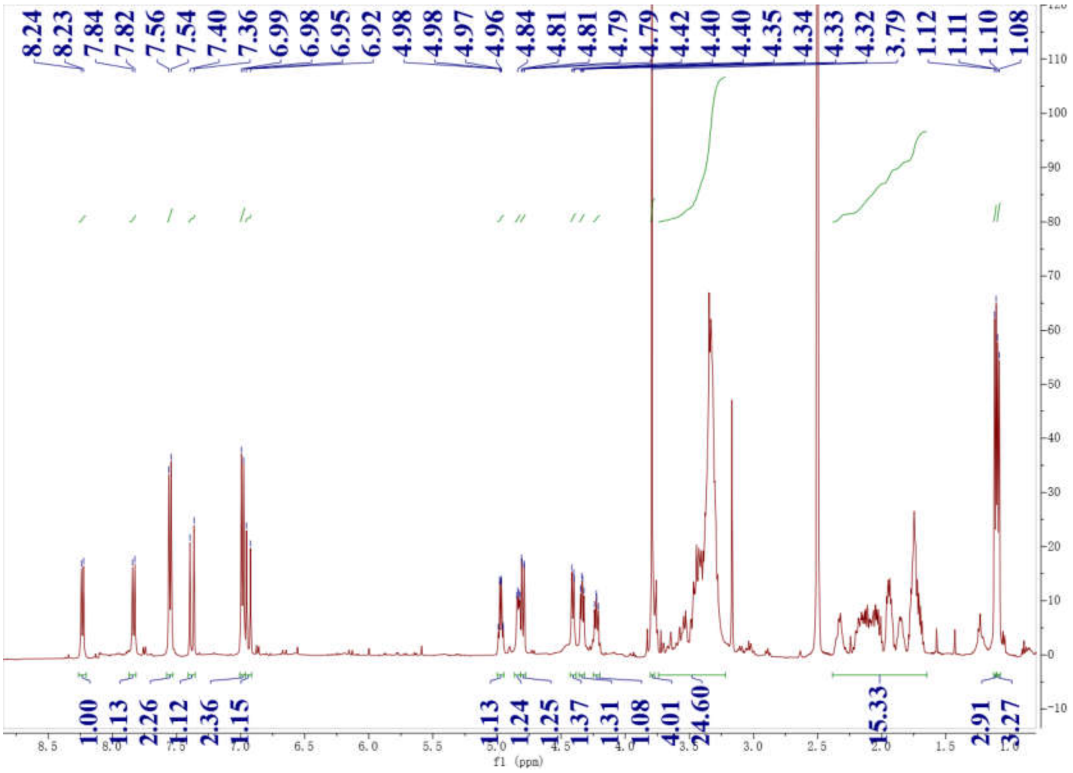

Figure S29: ^{13}C NMR spectrum of **5** in DMSO-*d6*.

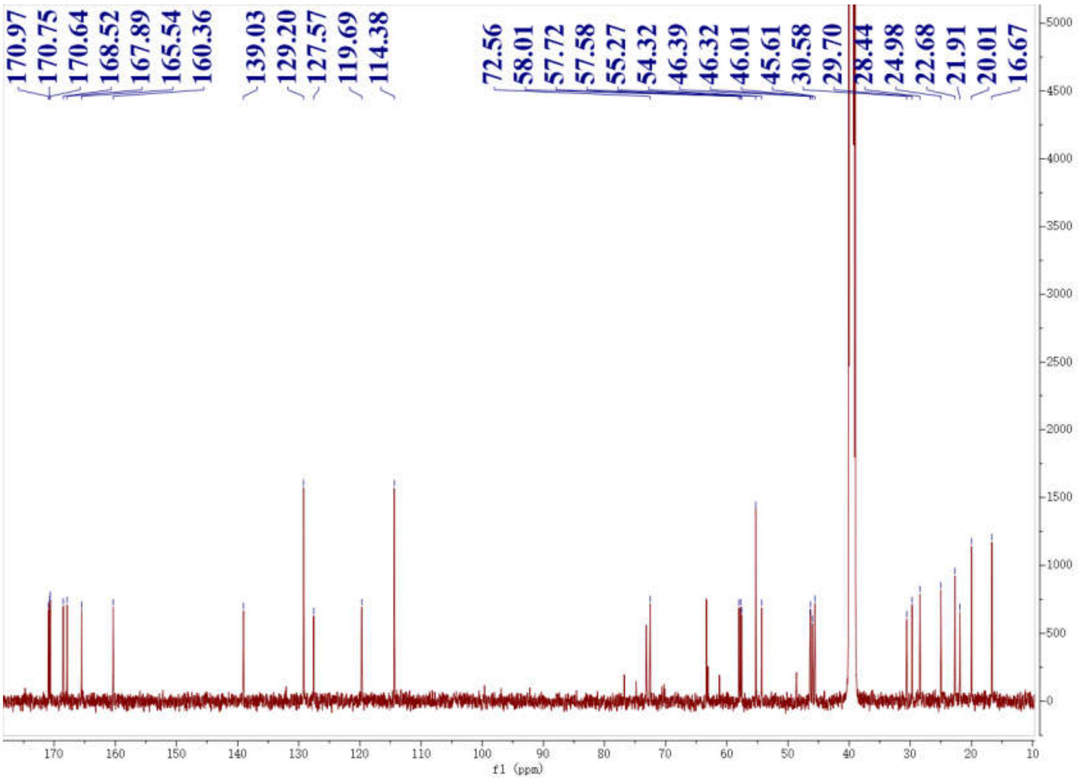

Figure S30: Acetylcholinesterase Inhibition Data for the Positive Control and Compounds 1–5.

Compound	aspertide E (**1**)	aspertide F (**2**)	aspertide C (**3**)	aspertide D (**4**)	aspertide A (**5**)	Background(B)	Positive Control(Tacrine)
	2.256	2.111	2.219	2.273	2.203	2.880	0.968
A	2.190	2.281	2.259	2.422	2.324	2.887	0.985
	2.311	2.178	2.191	2.190	2.332	2.901	
Mean Value(A)	2.252	2.190	2.223	2.295	2.286	2.889	0.977
	0.191	0.189	0.187	0.175	0.177	0.230	0.194
A_0	0.175	0.184	0.184	0.186	0.174	0.241	0.193
	0.183	0.199	0.182	0.175	0.173	0.235	
Mean Value A_0	0.183	0.191	0.184	0.179	0.175	0.235	0.194
$A-A_0$	2.069	1.999	2.039	2.116	2.112	2.654	0.783
Inhibition Rate(%)	22.03	24.67	23.19	20.26	20.43		70.52

Figure S31: Neuraminidase Inhibition Data and Standard Curve for the Compounds 1–5.

Standard Curve of the Positive Control						
Inhibition Rate(%)	100	80	75	50	25	0
Volume of Neuraminidase (x;μL)	0.01	1	2	5	7.5	10
Fluorescence Date(y)	481899	1978976	2906507	4396300	6436983	7530605
Fluorescence Date(y)	0.481899	1.978976	2.906507	4.3963	6.436983	7.530605
			The fluorescence readings were divided by 1,000,000 to simplify calculations.			
Compound	aspertide E (1)	aspertide F (2)	aspertide C (3)	aspertide D (4)	aspertide A (5)	
Fluorescence Date	5954678	6106990	5300482	5311040	5531039	
Fluorescence Date 2	5660873	6390438	5449165	4948912	5180730	
Fluorescence Date(y)	5.374823	5.355884	5.129976	5.80775	6.248714	
Volume of Neuraminidase (x;μL)	6.9988879	7.65274911	6.35694395	5.993884935	6.32886121	
Inhibition Rate(%)	30.01	23.47	36.43	40.06	36.71	

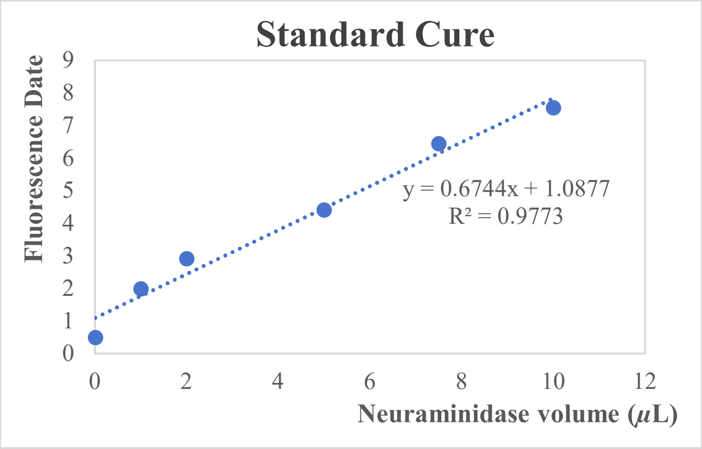

ITS sequence of the strain *Aspergillus* sp. SCSIO 41443

CAAGGTTTCCGTAGGTGAACCTGCGGAAGGATCATTACCGAGTGTAGGGTTCCTAGCGAG
CCCAACCTCCCACCCGTGTTTACTGTAACCTTAGTTGCTTCGGCGGGCCCGCCTTTAAGGC
CGCCGGGGGGCATCAGCCCCCGGGCCCGCGCCCGCCGGAGACACCACGAACTCTGTCTGA
TCTAGTGAAGTCTGAGTTGATTGTATCGCAATCAGTTAAAACTTTCAACAATGGATCTCTT
GGTTCCGGCATCGATGAAGAACGCAGCGAAATGCGATAACTAGTGTGAATTGCAGAATTC
CGTGAATCATCGAGTCTTTGAACGCACATTGCGCCCCCTGGTATTCCGGGGGGCATGCCTG
TCCGAGCGTCATTGCTGCCCATCAAGCACGGCTTGTGTGTTGGGTCGTCGTCCCCTCTTCG
GGGGGGACGGGCCCCAAAGGCAGCGGCGGCACCGCGTCCGATCCTCGAGCGTATGGGGC
TTTGTCACCCGCTCTGTAGGCCCGGCCGGCGCTTGCCGAACGCAAAACAACCATTCTTTCC
AGGTTGACCTCGGATCAGGTAGGGATACCCGCTGAACTTAAGCATATAAANNNGGGGG
